# CpG island erosion, polycomb occupancy and sequence motif enrichment at bivalent promoters in mammalian embryonic stem cells

**DOI:** 10.1038/srep16791

**Published:** 2015-11-19

**Authors:** Anna Mantsoki, Guillaume Devailly, Anagha Joshi

**Affiliations:** 1 Division of Developmental Biology, The Roslin Institute and Royal (Dick) School of Veterinary Studies, University of Edinburgh, Easter Bush Campus, Midlothian, EH25 9RG, UK

## Abstract

In embryonic stem (ES) cells, developmental regulators have a characteristic bivalent chromatin signature marked by simultaneous presence of both activation (H3K4me3) and repression (H3K27me3) signals and are thought to be in a ‘poised’ state for subsequent activation or silencing during differentiation. We collected eleven pairs (H3K4me3 and H3K27me3) of ChIP sequencing datasets in human ES cells and eight pairs in murine ES cells, and predicted high-confidence (HC) bivalent promoters. Over 85% of H3K27me3 marked promoters were bivalent in human and mouse ES cells. We found that (i) HC bivalent promoters were enriched for developmental factors and were highly likely to be differentially expressed upon transcription factor perturbation; (ii) murine HC bivalent promoters were occupied by both polycomb repressive component classes (PRC1 and PRC2) and grouped into four distinct clusters with different biological functions; (iii) HC bivalent and active promoters were CpG rich while H3K27me3-only promoters lacked CpG islands. Binding enrichment of distinct sets of regulators distinguished bivalent from active promoters. Moreover, a ‘TCCCC’ sequence motif was specifically enriched in bivalent promoters. Finally, this analysis will serve as a resource for future studies to further understand transcriptional regulation during embryonic development.

Embryonic stem (ES) cells have the unique ability to self-renew indefinitely as well as to differentiate in response to internal as well as external stimuli^1^. These two properties of ES cells pose specific constraints on the genome, as self-renewal requires maintenance of cellular memory that specifies its pluripotent capacity, while differentiation potential requires pluripotent ES cells to be highly plastic to enter any one distinct differentiation pathway. While the pluripotent state of ES cells is controlled through a network of core transcription factors^2^, emerging data point to a key role for epigenetic mechanisms such as chromatin dynamics and histone modifications in pluripotency^3^. Histone proteins and their post-translational modifications define the chromatin status of a cell and are correlated with the transcriptional status of genes. Mono-methylation of lysine 4 of histone protein 3 (H3K4me1) and acetylation of lysine 27 of histone protein 3 (H3K27ac) mark active enhancers while H3K4me3 and H3K27me3 mark active and repressed promoters, respectively^4^. Other epigenetic marks are also associated with promoters and enhancers. For example, H4K16 acetylation marks active genes and enhancers in ES cells^5^. Set/MLL histone methyltransferases, the mammalian homologues of the trithorax group proteins (trxG), catalyse the H3K4me3 marks and Polycomb (PcG) group proteins catalyse H3K27me3. Both complexes are thought to regulate expression of important differentiation and developmental genes^6^,^7^. These two chromatin modifications previously thought to be mutually exclusive were observed co-existing on promoters in murine ES cells and were named ‘bivalent’ promoters^8^. Bivalent genes are typically silenced or expressed at a very low level in ES cells, and by the presence of both active and repressive marks, are thought to be poised for activation or repression during the differentiation process^9^,^10^. Bivalent genes in ES cells either lost the H3K27me3 mark and were expressed, or lost H3K4me3 and were silenced when differentiated into the neuronal lineage^9^. Upon receiving endoderm differentiation signals, the bivalent BRACHYURY and NODAL promoters in human ES cells were unilaterally resolved to activation of the associated genes by losing H3K27me3^11^.

Bivalency of chromatin has therefore become an important property to investigate the functional relevance of a gene through development, and the presence of bivalent genes in human and mouse ES cells has been validated by many studies independently^9^,^12^,^13^,^14^,^15^. Here we performed a systematic identification and characterisation of bivalent genes and their functions by integrating all publicly available pairs (H3K4me3 and H3K27me3 measured on the same samples) of ChIP sequencing datasets in human and mouse ES cells, and identified and characterised a set of 4,979 and 3,659 high–confidence (HC) bivalent promoters respectively.

## Material and Methods

### HC bivalent promoter detection

ChIP sequencing raw data for H3K4me3 and H3K27me3 histone marks in murine and human ESCs was obtained from Gene Expression Omnibus (GEO)^16^ and Roadmap Epigenomics^17^ databases. Detailed description of the high confidence bivalent promoter detection method is provided in supplementary methods. In short, after mapping the reads to mm10 or hg19 genomes peaks were called in each sample (using the input control whenever provided) using SICER^18^. The peaks were then intersected with the 38,922 mouse (Gencode M2) and 57,818 human transcription start sites (TSSs) (Gencode 19) from GENCODE^19^, which include both protein coding as well as non-coding genes. H3K4me3 marks promoters with sharp peaks, while H3K27me3 occupies wide domains over the entire gene body. To account for these differences, we defined a −1000 to +2000 bp region around the TSS as the promoter region. The chromatin status of promoters for each sample is summarised in tables S1 and S2. Bivalent promoters identified in over 70% of samples were defined as high confidence.

### Human and mouse comparison

The one2one orthologous regions between human and mouse (16,639 genes) were obtained from Ensembl BioMart^20^. For comparative analysis between species, we used bivalent regions for each species and their corresponding regions in other species (human/mouse) using the UCSC liftOver tool^21^.

### Clustering using PRC components and RNA PolII

For the clustering of bivalent promoters into four groups, we gathered chip sequencing data for three different forms of RNAPII: RNAPIIS5P, RNAPIIS7P and 8WG16^22^, PRC2 components: Suz12^23^, Jarid2^24^, and PRC1 subunits: Cbx7 and Ring1b^23^ in murine ES cells and four groups were identified using k-means clustering from seqMINER tool^25^.

### CpG density

We calculated the CpG density as the ratio of observed to expected CpG counts^26^ in 100 bp window from −5 to +5 Kb around the TSS.

### Transcription and epigenetic factor ChIP-seq

For identification of factors enriched at bivalent and H3K4me3 promoters, we used data from 49 and 99 ChIP-seq experiments for several factors in human and mouse embryonic stem cells respectively^27^ and the significance of overlap was calculated using a hypergeometric test.

### Gene expression

RNA sequencing data in murine ES cells^28^ as well as from 63 single cells^29^ were used to show that bivalent promoters are lowly expressed compared to H3K4me3 only promoters. We collected differentially expressed gene lists after over expression of 54 factors and deletion of 37 factors individually in murine ES cells^30^.

### Sequence motif and functional enrichment

The sequence motif enrichment analysis was performed with the command findMotifs.pl from HOMER^31^. We conducted gene ontology functional analyses for the bivalent promoters using DAVID^32^ and AMIGO^33^.

## Results

### High-confidence bivalent promoters in human and mouse ES cells are enriched for developmental regulators

**Figure 1:**
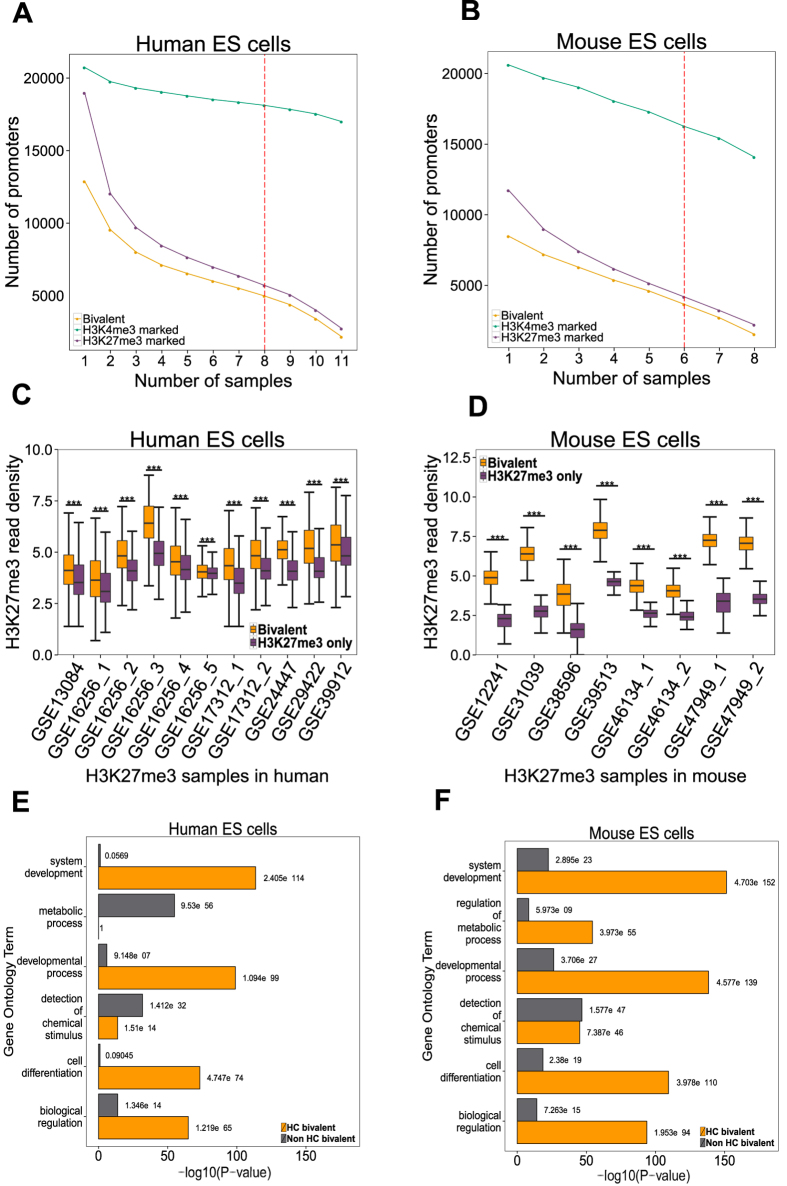
Identification of high confidence bivalent promoters in human and mouse ES cells. (**A**) The number of H3K4me3 (green), H3K27me3 (purple) and bivalent (yellow) promoters detected in ‘n’ or more samples (x axis) in human (left) and mouse ES cells (right). The red dotted line represents the cut off used to define high-confidence bivalent promoters. (**B**) H3K27me3 read density (in log scale) at bivalent and H3K27me3 only promoters in each sample designated by their GEO accession number (x axis) in human (left) and mouse (right) ES cells (***P-value < 10^−4^). (**C**) Gene Ontology terms enriched in HC bivalent promoter list (yellow) or non HC bivalent promoter list (grey) in human (left) and mouse (right) ES cells with their corresponding P-value.

Bivalent promoters are distinguished by the presence of both H3K4me3 and H3K27me3 modifications and are thought to mark developmental regulators in ES cells. To determine a robust set of bivalent promoters, we collected 11 pairs (i.e., generated by the same lab using same ES cell samples) of H3K4me3 and H3K27me3 ChIP sequencing (ChIP-seq) datasets for human ES cells and 8 pairs for mouse ES cells from the Gene Expression Omnibus (GEO) database and the Roadmap Epigenomics Project (Tables S1,S2 and Methods). After aligning reads to the respective genomes, peaks were called in each dataset using SICER^18^ and were overlapped with 57,818 human promoters from GENCODE 19^19^ and 38,922 murine promoters from GENCODE M2^19^. The number of H3K4me3 marked promoters across data sets was highly consistent (human: mean 18,632.55 relative SD 2.8%, mouse: mean 17,554.25 relative SD 11%), in contrast to the number of H3K27me3 marked promoters (human: mean 7,523.45 relative SD 37%, mouse: mean 6,128.75 relative SD 35%) (Tables S3 and S4). Moreover, the same promoters were consistently identified as H3K4me3 marked across samples, as demonstrated by incrementally intersecting the peaks from multiple datasets (Fig. 1A, green curve). In contrast, the H3K27me3 marked promoters (Fig. 1A, purple curve) varied across datasets, strongly influencing the number of bivalent promoters detected (Fig. 1A, yellow curve). Assigning a bivalent status to a promoter is therefore largely subject to H3K27me3 peak identification on the promoter. Over 85% of H3K27me3 marked promoters in both human and mouse were bivalent promoters (Fig. 1A, Tables S5 and S6). Thus, we reconfirm that bivalency at the H3K27me3 marked promoters is rather a rule than an exception^15^. The sequencing depth across samples varied from 14 million to over 100 million which might contribute to the variation of bivalent promoter detection in individual datasets. Indeed there was a high correlation between the number of reads and number of peaks across murine datasets (for H3K27me3 Pearson’s correlation coefficient (***r) ***= 0.75, for H3K4me3 ***r ***= 0.84), but not across human datasets (for H3K27me3 ***r ***= −0.20, for H3K4me3 ***r ***= 0.14). There are other factors contributing to the variation between samples, for example ES cells were grown in diverse culture conditions, and using different cell lines as well as various antibodies across datasets (Tables S1 and S2). We therefore defined bivalent promoters identified in more than 70% of the datasets (eight or more human datasets and six or more murine datasets) as high confidence (HC), resulting in 4,979 human and 3,659 murine HC bivalent promoters (Fig. 1A). Eight HC bivalent regions were validated by ChIP qPCR for the presence of H3K27me3 modification^9^ (Table S11). Adding or removing a sample in defining HC promoters did not change the key findings of the downstream analysis (see supplementary methods and Figure S1). There was no strong correlation between the fraction of HC bivalent promoters detected in a sample and the sequencing depth of that sample for both histone modifications (Pearson’s Correlation: Human: r = −0.34 H3K27me3, r = −0.38 H3K4me3, Mouse: r = 0.35 H3K27me3, r = −0.112 H3K4me3) (Figure S2).

HC bivalent promoters had higher H3K27me3 read density than H3K27me3-only promoters in any individual dataset (Student’s t-test, P-value < 0.0001) (Fig. 1B and S7), while H3K4me3 read density at HC bivalent promoters was lower than at H3K4me3-only promoters (Student’s t-test, P-value < 0.0001) (Figures S3 and S8). To test whether integration of multiple samples simply resulted in selecting the peaks with the strongest signal (peak height) from individual H3K27me3 samples, we selected the top (highest H3K27me3 signal) 4,979 human and 3,659 murine bivalent promoter peaks in each dataset and calculated the overlap with HC bivalent promoters. Less than 2/3^rd^ of H3K27me3 top promoters in any individual dataset overlapped with HC bivalent promoters (Figure S4).

We also checked whether the peaks of H3K27me3 and H3K4me3 modifications were present at the same genomic location within a promoter region and found that over 95% of H3K27me3 and H3K4me3 peaks overlapped in each pair of samples at HC bivalent promoters. Both chromatin modifications were indeed present at the same genomic location (Figure S5). We compared the functional enrichment between high-confidence and non-high-confidence (detected as bivalent in less than 70% of datasets) bivalent promoters and found that only the high-confidence promoters were strongly enriched for processes such as ‘cell differentiation’ and ‘system development’ (Fig. 1C). Interestingly, metabolic processes were enriched in murine but not human HC bivalent promoters.

In summary, by integrating data from multiple studies we identified HC human and murine bivalent promoters, which could not be identified by simply selecting the top peaks from individual samples. The HC bivalent promoters were highly enriched for developmental regulators compared to non-HC bivalent promoters.

### High-confidence bivalent promoters are marked by PRC1, PRC2 and RNA polymerase II

**Figure 2:**
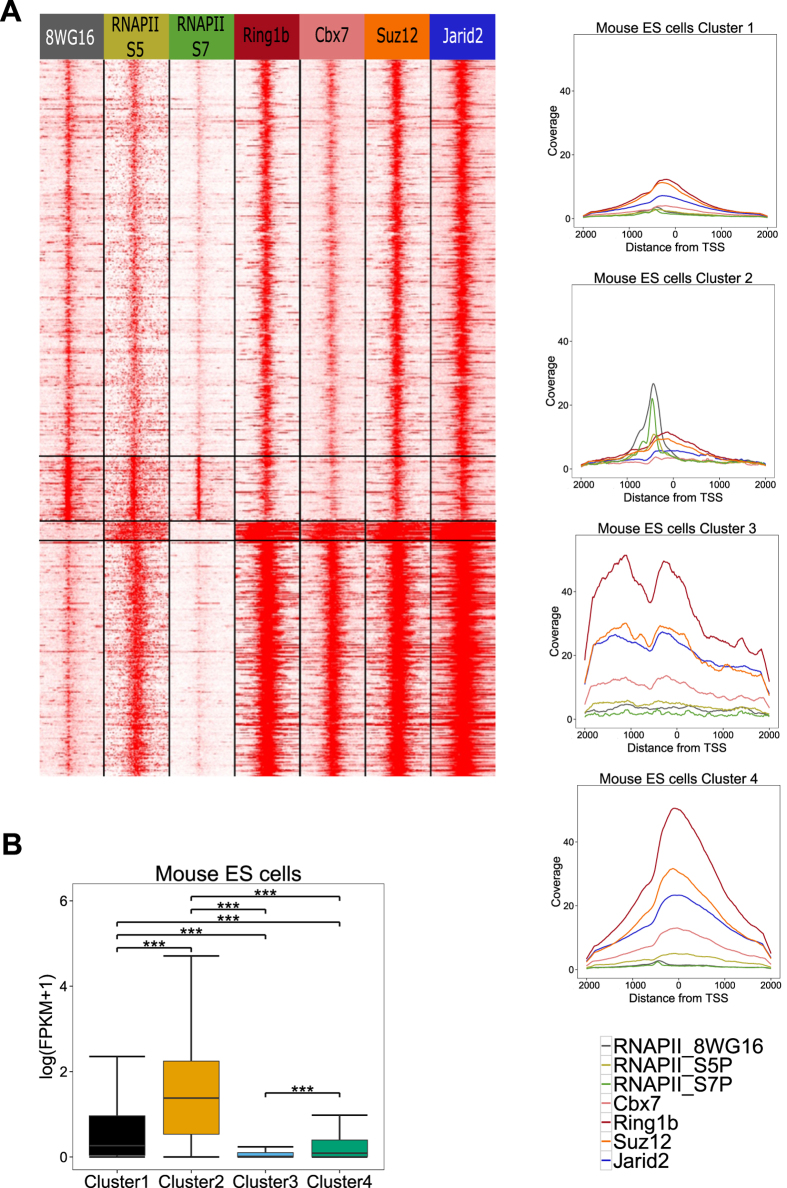
Four groups of HC bivalent promoters with distinct biological features. (A) HC bivalent promoters in murine ES cells classified in four subgroups based on occupancy of PRC1 components (Ring1b, Cbx7), PRC2 (Suz12), Jarid2 and RNA polymerase II (Ser7P, Ser5P, 8WG16). Each line represents one single promoter while color code summarizes ChIP-seq read densities, from −5kb to +5Kb around TSS. For each cluster, mean read coverage around TSS is shown on the right. (**B**) Expression levels in mouse ES cells using RNA sequencing data for each of the four clusters. FPKM: Fragment per kilo-base per million (***P-value < 10^−4^).

Bivalent promoters are known to show variation in their levels of occupancy by RNA polymerase II^22^ and PRC complexes^12^ . To further characterize HC bivalent promoters, we gathered ChIP-seq data in murine ES cells for various forms of RNAPII phosphorylated in different residues (RNAPIIS5P and RNAPIIS7P) as well as RNAPII8WG16 (an antibody that recognizes mostly unphosphorylated PolII)^22^, together with ChIP-seq data for the SUZ12, a subunit of PRC2, responsible for catalysing the histone modification H3K27me3, the RING1B and CBX7 subunits of PRC1^23^, responsible for catalysing H2Aub1 and for compacting chromatin, and Jarid2^24^. Jarid2 is a co-factor of PRC2 and is methylated by PRC2 which in turn promotes PRC2 activity^34^. All HC bivalent promoters were marked by both PRC1 and PRC2 components albeit at different levels (Fig. 2A).

HC bivalent promoters could be classified in four distinct clusters based on the presence of PRC1 components and forms of RNAPII (Fig. 2A). The first two clusters had low PRC1 (Ring1b) levels and high RNAPII (8WG16) levels compared to clusters 3 and 4. The second cluster distinguished from the first cluster by the presence of RNAPII (8WG16 and S5P) modifications as a sharp peak on the promoter. The second cluster consisted of the only group of bivalent promoters marked with RNAPII (S7P). This cluster was enriched for genes involved in metabolic processes. The third and fourth clusters were marked by strong PRC1 (Ring1b), PRC2 (Suz12) and RNAPII (S5P) modifications. Cluster 3 and 4 were distinguished based on the fact that PRC components formed wide domains on cluster 3 and narrow peaks on cluster 4 promoters. Cluster 3 promoters were enriched for regulation of transcription (P value < 10^−51^) while cluster 4 promoters were enriched for developmental functions such as organ morphogenesis (P value < 10^−27^). Cluster 3 promoters contained transcription factors important for specific lineages like haematopoiesis factors Gfi1 and Meis1, whereas cluster 4 contained multiple members of transcription factor families controlling development such as winged helix/forkhead box (Fox) and Hox families.

We noted that bivalent promoters could be distinguished into two groups based on PRC1 occupancy: PRC1 low (cluster 1 & 2) and PRC1 high (cluster 3 & 4). Ku *et al.* (12) suggested that PRC1 was absent in our PRC1 low bivalent promoter (Figure S11). Ring1b ChIP sequencing at higher sequencing depth confirms that all bivalent promoter are bound by PRC1 albeit at different levels. The PRC1 high group separated into two distinct groups each enriched for a distinct functional category, namely cluster 3 for transcription factors and cluster 4 for developmental controllers. Based on RNA PolII occupancy, PRC1 low consisted of two distinct gene sets: PolII-low (S7P) and PolII-high (S7P). The difference in chromatin signature of these two clusters was also reflected in the expression level namely PolII-high (cluster 2) promoters were expressed at higher levels than PolII-low or cluster 1 promoters (Kruskal-Wallis test P-value < 0.0001) (Fig. 2B).

In summary, all HC bivalent promoters are occupied by components of both PRC1 and PRC2. There exists a distinct set of metabolic genes (cluster 2) which though bivalently marked has RNAPII (S7P) and is expressed at a higher level than other bivalent genes.

### Bivalent promoters are lowly expressed and highly sensitive to perturbations in ES cells

**Figure 3:**
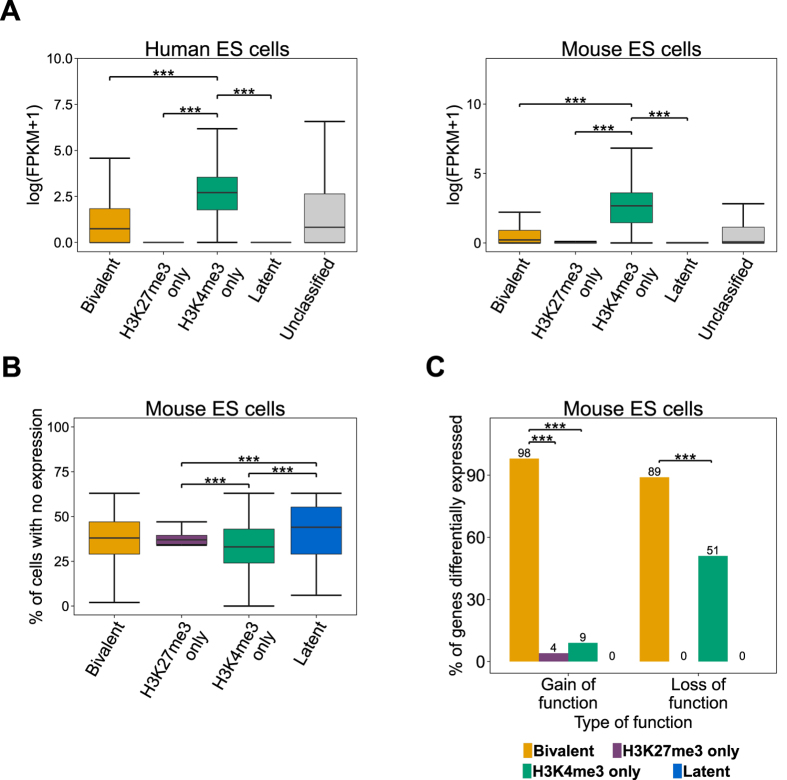
Bivalent promoters are lowly expressed in ES cells and are more likely to be differentially expressed upon perturbation. (**A**) Expression levels according to human (left) or mouse (right) ES cells RNA-seq for HC bivalent promoters (yellow), promoters marked with H3K27me3 only (purple), promoters marked with H3K4me3 only (green), latent promoter (blue), or unclassified promoters (grey, see text) (***P-value < 10^−4^). (**B**) From single cell RNA-seq data of mouse ES cells, percentage of cells non expressing the lowly expressed genes (i.e. FPKM < 4) was computed for different classes of promoters (bivalent, H3K27me3 only, H3K4me3 only and latent) (***P-value  < 10^−4^). (**C**) HC bivalent promoters are hypersensitive to changes in the transcription network perturbation. Differentially expressed gene lists were collected from studies overexpressing one of 54 factors (gain of function) or down-regulation of one of 37 factors in ES cells. Percentage of significantly overlapping (P value < 1e-3) bivalent, H3K27me3 only, H3K4me3 only and latent genes with differentially expressed in at least one of the experiments is represented (***P-value < 10^−4^).

RNA polymerase II (PolII) may be present but stalled at the promoters of bivalent genes and short (abortive) transcripts may be detected at their promoters^35^. To check whether bivalent genes indeed show a low or leaky expression, we collected RNA sequencing data for murine^28^ and human^36^ ES cells and calculated the mean expression level for the following categories of promoters: We classified promoters into four HC groups (Table S9 and S10) depending on the presence or absence of one or both chromatin modifications in over 70% of samples as bivalent promoters, promoters marked only with H3K27me3 (H3K27me3-only), promoters marked only with H3K4me3 (henceforth called ‘active’) and latent promoters (unmarked for H3K27me3 and H3K4me3). Promoters that belonged to any of the previous four categories in less than 70% of the samples, and thus were not considered in that category were marked as unclassified. In human and mouse ES cells, most active promoters were expressed at higher levels than bivalent promoters, and latent promoters were mostly not expressed (FPKM = 0) (Kruskal-Wallis test, P-value < 0.0001) (Fig. 3A). Low expression can result from two scenarios: either a gene is expressed at low levels in most cells or few cells express a gene while others do not. To determine whether lowly expressed genes in the four groups can be classified into one of the two scenarios, we downloaded single cell RNA sequencing data for 63 mouse ES cells^29^. Lowly expressed (i.e. FPKM < 4, or log(FPKM) < 1.4) active promoters were expressed in a similar number of single cells as lowly expressed bivalent promoters (Kruskal-Wallis test, P-value > 0.05) (Fig. 3B) demonstrating that single cell gene expression data cannot distinguish between bivalent and active lowly expressed genes.

As bivalent genes are thought to be poised for activation or repression, we hypothesised that these genes might be more likely to be differentially expressed upon perturbation of ES cells. We therefore used a collection of differentially expressed genes upon deletion or over-expression of 91 transcription and epigenetic factors in mouse ES cells, and found that 98% of differentially expressed gene sets by the overexpression of at least one TF significantly overlapped (Hypergeometric test, P value < 1e-3) with bivalent genes, and 89% differentially expressed gene sets by the down-regulation of at least one TF (Fig. 3C). To check whether this is a property of bivalent genes or lowly expressed genes in general, we also calculated the overlap of active and latent lowly expressed genes with the differentially expressed gene sets upon transcription and epigenetic factor perturbation. We confirmed that bivalent genes are highly susceptible to perturbations compared to active or latent lowly expressed genes (Kruskal-Wallis test, P-value < 0.001) (Figure S6).

### Over 50% of bivalent promoters maintain their chromatin status as well as gene expression profile across species

**Figure 4:**
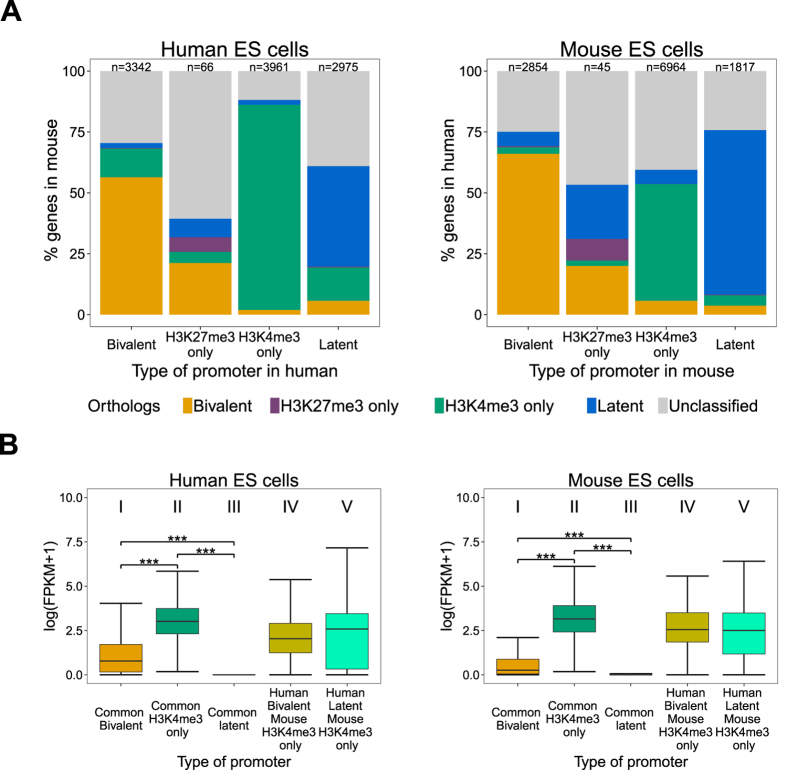
Over 50% of bivalent promoters maintain their chromatin status as well as gene expression profile across species. (**A**) Overlap of high confidence (HC) H3K4me3 only (green), H3K7me3 only (purple), bivalent (yellow) and latent (blue, absence of both H3K4me3 and H3K7me3 modifications) in human ES cells with the corresponding categories in mouse ES cells (left), and vice versa (right). Grey: Unclassified promoters (see text). (**B**) Expression levels in human (right) and mouse (left) ES cells using RNA sequencing data for each of the five groups of orthologous genes identified in (**A**) (***P-value < 10^−4^).

To perform a systematic comparison of chromatin status between human and mouse promoters in ES cells, we used 16,639 one-to-one orthologous genes between the two species^20^. We classified orthologous promoters into four HC groups – active (H3K4me3-only), H3K27me3-only, bivalent and latent. Promoters that did not belong to any of the previously mentioned groups were designated as ‘unclassified’. We confirmed that HC H3K27me3-only and active promoters indeed had low or no other chromatin modification (Figures S7 and S8). We then calculated the overlap of the five groups across species (Fig. 4A). Over 40% of murine orthologous promoters (n = 6964) contain an activating mark (H3K4me3-only), in contrast to only 24% of human orthologous promoters (n = 3961). There was a 47% overlap of murine active promoters with human active promoters; while 84% of human active promoters overlapped with murine active promoters i.e. most active promoters in human are also active in mouse but not vice versa. Bivalent promoters constitute 17% (n = 2854) and 20% (n = 3342) of mouse and human orthologous genes respectively. 66% of murine bivalent promoters are also bivalent in human and 56% of human bivalent promoters are bivalent in mouse. The promoters with the H3K27me3-only modification form a very small fraction of orthologous promoters reaching merely 0.2% (n = 45) and 0.3% (n = 66) in mouse and human respectively. About 20% of H3K27me3-only promoters in one species are bivalent in the other species. Conserved bivalent promoters were enriched for functional categories developmental protein (P value <10^−71^) and transcription factor activity (P value < 10^−65^); whereas species-specific promoters were not enriched for the two above terms (Table S7). Specifically, the mouse-specific bivalent promoters were enriched for membrane (P value < 10^−16^) and glycoprotein (P value < 10^−13^) and the human-specific for plasma membrane part (P value < 10^−5^) and alternative splicing (P value < 10^−3^).

To check whether the chromatin status across species is reflected in the gene expression status, we focused on five groups of promoters (Fig. 4B): three groups (I, II and III) with conserved chromatin status and two groups with divergent chromatin status (IV and V) across species. The gene expression profiles of conserved chromatin groups across species were also conserved. Specifically, active promoters (II) were expressed at higher level than bivalent promoters (I) which in turn were expressed at higher level than latent promoters (III) in both human and mouse ES cells (Kruskal-Wallis test, P-value < 0.0001) (Fig. 4B). The divergence of chromatin status promoters across species was not reflected in the gene expression level. For example the orthologous promoters with bivalent status in human and active status in mouse (IV) were expressed at intermediate levels between active (II) and bivalent promoters (I) in both species (Fig. 4B).

### Bivalent promoters are CpG rich while H3K27me3-only promoters are CpG poor

**Figure 5:**
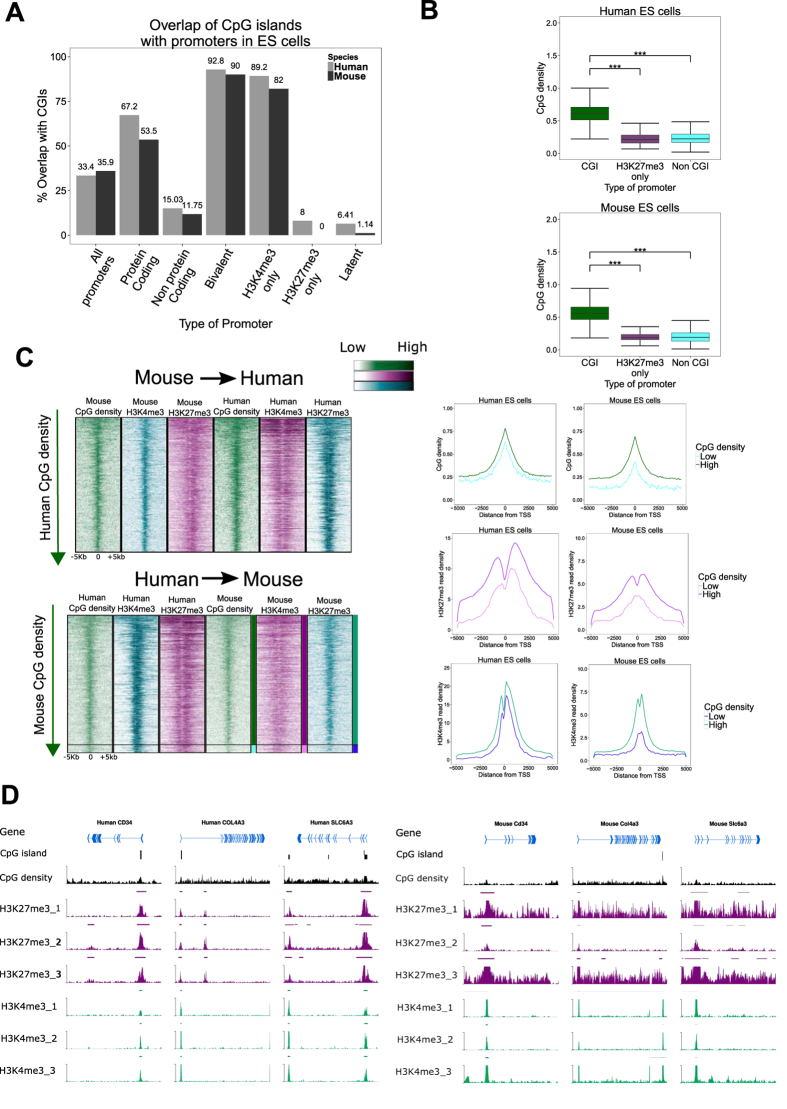
Bivalent promoters are CpG island rich while H3K27me3 only are CGI poor. (**A**) Percentage of promoters overlapping with one or more CpG island in human (grey) or mouse (black). (**B**) CpG ratio at H3K27me3 only promoters is similar to non-CGI promoters in human (top) and mouse (bottom) ES cells (***P-value < 10^−4^). (**C**) Relationship between CpG density, H3K27me3 modification and H3K4me3 modification in human and mouse ES cells. There is a loss of human CGI promoters in mouse (bottom, below marked black line) but no loss of mouse CGI promoters in human (top). This loss is linked with decreasing H3K4me3 and H3K27me3 in mouse as compare with human. Left panels indicate mean CpG densities, mean H3K27me3 read densities and mean H3K4me3 densities in human and mouse. (**D**) Exemplar murine promoters where CGI loss on promoters does not correspond to the loss H3K27me3 modification. These promoters despite losing CGI keep bivalent promoter status in murine ES cells.

As shown in the first section, the bivalent status of promoters is primarily determined by the detection of an H3K27me3 modification (Fig. 1A). CpG islands (CGIs) have been implicated in polycomb recruitment and therefore H3K27me3 modification^37^,^38^,^39^. CGIs are CpG-rich genomic regions and are sites of transcription initiation^40^. CGI promoters are silenced by either DNA methylation or polycomb group proteins with approximately a fifth of CGI promoters accounting for bivalent promoters in ES cells^12^. About 35% of all GENCODE genes in both human and mouse overlapped with at least one CGI. When only protein coding genes were considered, this overlap increased to 67% for human and 54% for mouse (Fig. 5A). Mouse promoters in most categories showed lower overlap with CGIs than human promoters (Fig. 5A). 89% of human active (H3K4me3-only) as well as 82% of murine active promoters contained at least one CGI (Fig. 5A).

Over 90% of our HC bivalent promoters in ES cells in both species overlap with at least one CGI region, whereas only 8% (37 of 397) of human H3K27me3 only promoters contained a CGI and no mouse H3K27me3 only promoters (none of 152) contained a CGI (Fig. 5A). Previously CGIs have been associated with H3K27me3 modification in mammalian ES cells^41^,^42^, but our results show that this is the case for bivalent promoters but not for H3K27me3 only promoters. We confirmed that the lack of CGIs on active promoters is not due to the CGI detection threshold and that the CpG density at repressed promoters is indeed significantly lower than at CGIs (Kruskal-Wallis test, P-value < 0.0001)(Fig. 5B). It has been proposed that a high density of un-methylated CpG is sufficient for vertebrate polycomb recruitment^42^. The fact that H3K27me3-only promoters are specifically CpG-poor (Fig. 5A,B), suggests that, although highly unmethylated CpG islands might be sufficient for polycomb recruitment, they might not be necessary.

The loss of H3K27me3 in rodents (mouse and rat) compared to human ES cells at many developmental genes has been associated with depletion of CGIs; mouse CGI erosion has been characterised at MYO1G, CLEC4G and MYF6 gene loci with corresponding H3K27me3 loss^41^. We performed a cross-species comparison of CpG density, H3K4me3 and H3K27me3 profiles of bivalent promoters (Fig. 5C). Indeed, about 5% of bivalent human promoters lost CGIs in mouse but not vice versa (indicated by black horizontal line). There was a high correlation between CpG density and H3K4me3 as well as H3K27me3 profiles within each species as well as across species (Fig. 5C), but the concordance between loss/gain of CGIs and H3K4me3 and/or H3K27me3 mark does not always hold true. Of 70 orthologous CpG-rich bivalent promoters in human where CGI was lost in mouse and analysed their chromatin status, only 18% of these promoters had clearly lost their H3K27me3 mark in mouse ES cells, of which half were classified as H3K4me3-only and the rest as latent in murine ES cells (Figure S9). Despite losing CGI on murine promoters, 20% of these orthologous promoters maintained a bivalent chromatin status including Col4a3, Cd34 and Slc6a3 (Fig. 5D).

In summary, the H3K27me3-only CpG-poor promoters demonstrate that polycomb recruitment does not only depend on CpG density. Although the CpG density largely correlates with H3K4me3 and H3K27me3 profiles across promoters, the loss of CGI on a promoter does not always imply a corresponding loss of the H3K4me3 and/or H3K27me3 modification on that promoter.

### Bivalent promoters are occupied by fewer transcription factors than active promoters and are specifically enriched in a ‘TCCCC’ sequence motif

**Figure 6:**
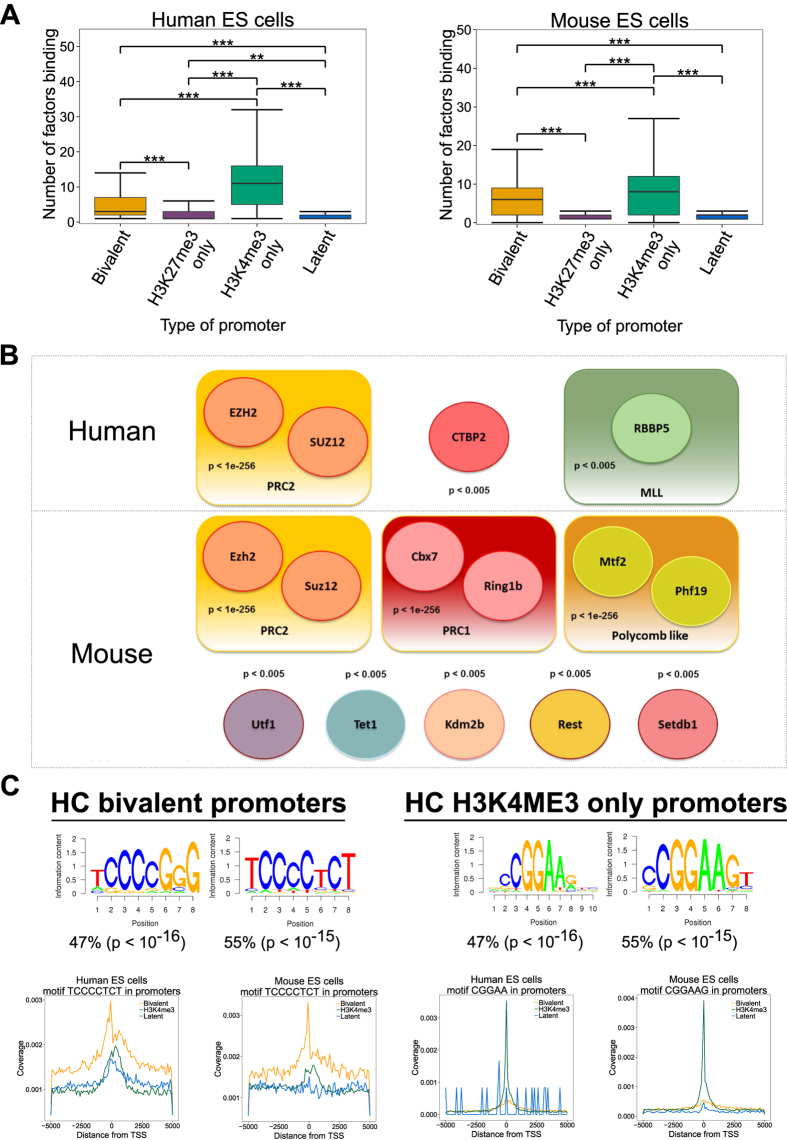
(**A**) ‘TCCCC’ sequence motif is specifically enriched in bivalent promoters. (**A**) The average occupancy of factors at HC H3K4me3-only promoters (green) is higher than at HC bivalent promoters (yellow) which is higher than at HC H3K27me3 only promoters (purple) and latent promoters (blue) in human (left) and mouse (right) ES cells (***equals to <10^−4^). (**B**) Transcription and epigenetic factors with statistically significant overlap with HC bivalent promoters from ChIP sequencing data for 49 in human (up) and 99 factors in mouse (down) ES cells. (**C**) A ‘TCCCC’ sequence motif is specifically enriched in HC bivalent promoters in both human and mouse ES cells. Similarly a ‘CGGAA’ motif is enriched HC H3K4me3 promoters in both human and mouse ES cells. Each motif was then mapped to the genome, and motif densities around TSSs of bivalent (black), H3K4me3-only (yellow) and latent (blue) promoters are shown in the left (human) and right (mouse) panels.

As both active (H3K4me3-only) and bivalent promoters are CpG-rich, we investigated possible modes of distinction between the two in ES cells. Voigt, Tee, and Reinberg^43^ proposed a model where the density of transcription factors at the promoters determines establishment of bivalent domains. Specifically, the model suggests that PcG proteins are inhibited from binding at active promoters by an abundance of transcription factors, while at promoter sites with a low occupancy of transcription factors, PcG proteins can easily be recruited at CpG islands to establish the H3K27me3 modification. To test this model, we used publicly available genome-wide TF and epigenetic modifier binding profiles (ChIP-seq data) in murine and human ES cells^44^ and calculated the number of transcription factors bound (TF density) at the four classes of promoters. Indeed the TF density decreases from active to bivalent to H3K27me3-only promoters in both human and mouse ES cells (Kruskal-Wallis test, P-value < 0.0001) (Fig. 6A).

To identify factors preferentially binding to bivalent promoters, we calculated the overlap between transcription and epigenetic factor binding sites (peaks) and bivalent promoters. Four out of 49 and eleven out of 99 factors characterised by ChIP-seq preferred bivalent promoters in human and mouse respectively (Fig. 6B). As expected, members of the PcG family were enriched at both human and mouse bivalent promoters (P value < 10^−256^). Moreover, the co-repressor c-terminal binding protein 2 (CTBP2), required for PcG recruitment in Drosophila^45^, and the RBBP5 (MLL subunit) were enriched at human bivalent promoters (P value < 0.005). The components of both PRC2 (Ezh2, Suz12) and PRC1 (Cbx7, Ring1b) together with two polycomb-like proteins (Mtf2, Phf9) were enriched at mouse bivalent promoters. Mtf2 and Phf19 recruit the PRC2 complex and are thought to silence transcriptionally active loci (H3K36me3) by recruiting H3K36me3 histone demethylases such as Kdm2b to further recruit PRC2 components for H3K27me3^46^,^47^,^48^. Accordingly, Kdm2b was also enriched at mouse bivalent promoters (P value < 10^−3^). Four other epigenetic regulators, Utf1, Tet1, Rest and Setdb1 were highly enriched at mouse bivalent regions. Utf1 (P value  < 10^−256^) was recently identified as a component of bivalent chromatin by acting as a buffer against full activation of bivalent genes^13^.

As expected, many TFs (33 out of 49 factors in human and 39 out of 99 factors in mouse) were enriched at active (H3K4me3-only) promoters. This included known regulators of pluripotency in ES cells such as Klf4, Esrrb, Oct4, Sox2, and Nanog (Table S8). Only two factors enriched in bivalent promoters, Kdm2b and Tet1, were also enriched at active promoters. All other factors showed preference to either bivalent promoters or active but not both. For example, C-Myc can stimulate Pol II elongation^48^ and was enriched in active promoters in both human and mouse ES cells but not in bivalent promoters.

The observation that some factors are enriched specifically at bivalent promoters suggests that sequence motifs specific to bivalent promoters may determine their binding. We performed *de novo* motif identification on bivalent promoters by providing active promoter sequences as background in HOMER software^31^ and found several AG-rich and GC-rich motifs specific to bivalent promoters (Figure S10). These resemble the sequence motifs of Jarid2^49^ and Utf1^13^ identified from ChIP-seq data. Interestingly, a ‘TCCCC’ sequence motif was enriched and found in about 50% of bivalent promoters in both human and mouse (Fig. 6C). This motif was not enriched in active promoters in either of the species (the number of repressed promoters was not large enough to perform a reliable *de novo* motif discovery). The ‘TCCCC’ motif was most similar to the known binding sequence of the Mzf1 transcription factor^50^. The Mzf1 promoter both in mouse and human ES cells is characterized as HC H3K4me3 only and belonged to the low expressed genes in our analysis. However, in recent Mzf1 ChIP-seq experiment performed in HEK293 cell line^51^, the “TCCCC” motif was not enriched in Mzf1 peak list (Table S9). When *de novo* motif enrichment was performed on active human and mouse promoters using bivalent promoter sequences as background, they were enriched for a ‘CGGAA’ motif found in 40% of the active promoter sequences, which was not enriched in bivalent promoters. This motif is the most similar to the known motif for Elk1 transcription factor (Fig. 6C).

In summary, bivalent promoters are bound by fewer transcription factors than active (H3K4me3-only) promoters, but more than H3K27me3 only and latent promoters. Active promoters were preferentially occupied by pluripotency factors. On the other hand, bivalent promoters were enriched for Polycomb factors as well as other chromatin modifiers. The factors enriched at bivalent promoters show very little overlap with the ones enriched at active promoters. These findings are consistent with the observed spatial segregation of transcriptional networks in ES cells where Nanog and Polycomb proteins were shown to occupy distinct nuclear spaces^52^. Finally, we identified a ‘TCCCC’ sequence motif specifically at bivalent promoters and a ‘CGGAA’ sequence motif at active promoters.

## Discussion

Bivalent chromatin domains bearing both H3K4me3 and H3K27me3 modifications have been shown to be a key feature of developmentally regulated genes in ES cells^8^,^9^,^13^,^14^,^15^. These domains are thought to be ‘poised’, with an ability to quickly become active (losing H3K27me3) or inactive (losing H3K4me3) during differentiation^9^,^53^. While many studies have produced ChIP-seq data for both H3K4me3 and H3K27me3 in ES cells in both humans^14^,^15^ and mice^9^,^13^, differences in species, ES growth conditions, ChIP protocols (shearing, cross link, antibodies used) and high throughput sequencing setup (with or without replicate, with or without input) have rendered a comparison across studies challenging. By systematic integration of available data, we identified robust lists of 4,979 and 3,659 high confidence bivalent promoters in human and mouse respectively. Since our work is using the data of previous studies using H3K4me3 and H3K27me3 ChIP-seq to define bivalency in ES cells, we are biased toward a confirmation of the original studies, as their data is integrated in our dataset. However our integrative approach (see methods) renders this analysis resistant to any outlier experiments. By cumulatively integrating the samples, it became evident that the detection of bivalency on promoters is dependent on the reliable detection of the H3K27me3 modification. Over 85% of H3K27me3 promoters were bivalent, i.e. they also had the H3K4me3 mark. This confirms that bivalency in ES cells is rather the rule than the exception. The three main chromatin states on promoters in ES cells are thus active, bivalent and latent (no mark). Correspondingly, active promoters were expressed, bivalent were lowly expressed and latent were mostly not expressed.

Bivalent promoters are thought to be poised for rapid activation or inactivation during differentiation^13^,^43^. To tease out whether the low expression at bivalent promoters is a result of some cells expressing the genes while others not, or the genes are expressed at low levels in most cells, we used single cell gene expression data. Bivalent genes were expressed in a similar number of single cells as lowly expressed active genes. It is therefore unlikely that bivalency is a result of mixture of cell populations in ES cells. Similarly, H3K27me3 read density was higher at HC bivalent promoters than at H3K27me3-only promoters, again arguing in disfavour of a mix-population model. The low transcription level can be interpreted as a “leaking” transcription rate, in the absence of a strong repressive chromatin environment. During development, these poised domains have been shown to resolve as either active (by losing the H3K27me3 mark) or inactive (by losing the H3K4me3 mark), and in some cases gaining DNA methylation^37^, depending on the cellular lineage. In agreement with this model, we have found that >90% of differentially expressed (either up-regulated or down-regulated) gene sets when any one of a set of 91 transcription factors was either overexpressed or knocked down in mouse ES cells were enriched for bivalent genes. This finding suggests that bivalent genes are hypersensitive to most perturbations of the regulatory network in ES cells.

We computed binding profiles of PRC components (PRC1 and PRC2) and various forms of RNA polymerase II at bivalent promoters in murine ES cells. All HC bivalent promoters were marked by Suz12, Jarid2, Ring1b and Cbx7. To note, the PRC2-only group defined by^12^ overlapped with PRC1-low clusters, the PRC1 signal detected due to higher sequencing depth in latter case (Figure S11). Thus all bivalent promoters were occupied by both PRC1 and PRC2. Accordingly, H2Aub showed enrichment at HC bivalent promoters (Figure S12). Recent studies have suggested that true bivalency is better associated with H2Aub than H3K27me3^22^. We note that H2Aub predominantly but not exclusively marks bivalent promoters (Table S10) as it also marks a fraction of H3K4me3-only expressed gene promoters (Figure S13). Based on PRC1 and RNA PolII occupancy, bivalent promoters grouped into four clusters. Clusters 1 and 2 had low PRC1 occupancy and high RNA PolII (8WG16) levels while clusters 3 and 4 were PRC-rich with low RNA PolII (8WG16) levels. Cluster 2 was enriched for metabolic genes and marked with RNA PolII (S7P) and cluster 2 genes were expressed at higher levels than the other three clusters. The bivalent promoters therefore consist of sub-groups of genes which at functional, epigenetic and transcriptional level are quite different from each other.

More than half of high-confidence bivalent promoters were conserved between human and mouse, suggesting the existence of a set of genes bivalently marked across most mammalian ES cells (Table S9 and S10). These genes were very highly enriched for transcription regulators and developmental factors, compared to the species specific bivalent promoters. On the other hand, divergence of epigenetic status across species did not imply divergence of gene expression i.e. promoters with bivalent chromatin status in human and active chromatin status in mouse did not have gene expression profiles similar to bivalent genes in human and active genes in mouse. Further analysis is necessary to understand whether the differences between mouse and human ES cells are indeed species-specific or developmental stage specific as human ES cells do not share the same developmental state as mouse ES cells^54^,^55^.

Since a high density of un-methylated CpG is sufficient for vertebrate polycomb recruitment^38^,^39^,^42^, it is assumed that the presence of CpG islands determines H3K27me3 modification. Over 90% of bivalent promoters contained a CpG island while few to none of the H3K27me3-only promoters had a CpG island. Wachter *et al.* (2014) recently suggested that bivalency is the default chromatin structure for CpG-rich, G+C-rich DNA^56^. The presence of H3K27me3 on CpG-poor promoters without H3K4me3 modification in ES cells (Figures S14 and S15) suggests mechanisms other than CpG islands for polycomb recruitment.

On bivalent promoters, the CpG density and H3K27me3 modification are highly correlated. By performing a cross-species comparison, a small fraction (~5%) of human CpG-rich HC bivalent promoters has the corresponding CpG eroded in the mouse genome, while no CpG-rich bivalent promoters in mouse are eroded in human. This erosion of CpG density was correlated with the loss of H3K27me3 and H3K4me3^41^. However, in about 20% of the cases, the CpG density loss in mouse compared to human did not correspond to a loss of H3K27me3. This reiterates the finding that CpG density might be sufficient but not necessary for H3K27me3 modification.

It is intriguing how bivalent domains are established in ES cells. Voigt *et al.*^43^ proposed a model where H3K4me3 marked promoters occupied by a low number of transcription factors allowed the establishment of H3K27me3 modification. Indeed, HC bivalent promoters were bound by fewer factors than active promoters in human and mouse ES cells. HC bivalent promoters were specifically enriched in ChIP-seq peaks for many members of the PRC1, PRC2 and MLL complexes as expected. We also found enrichment for several additional proteins known to be involved in recruiting these complexes, including CTBP2, Mtf2 and Phf19. Other factors frequently binding to HC bivalent promoters included Kdm2b, Utf1, Tet1, Rest and Setdb1. These factors are involved in establishing diverse epigenetic modifications suggesting the complex epigenetic regulation of these regions.

As active (H3K4me3-only) and bivalent promoters are both CpG rich, it is key to unravel the distinguishing factors between these two groups. *De novo* motif discovery at HC bivalent promoters identified a ‘TCCCC’ motif in both human and mouse ES cells which was not enriched at active promoters. This motif was present in about half of the HC bivalent promoters and is similar to the sequence motif of MZF1^50^, although this was not confirmed in recent MZF1 ChIP-seq experiment in HEK293 cell line^51^. Similarly, a ‘CGGAA’ motif was enriched specifically at active promoters and is similar to the sequence motif of ELK1. Further experiments are mandate to establish whether these sequence motifs indeed play a role at bivalent and active promoters, and if yes, through which factors? Characterising factors associated with these motifs will be the first step to study their functional relevance.

In summary, this meta-analysis revealed several novel aspects of bivalency in mammalian ES cells and will serve as a resource for future studies to further understand transcriptional regulation during embryonic development. Further work will be aimed at understanding how the HC bivalent promoters identified here are resolved in different cellular lineages during differentiation.

## Additional Information

**How to cite this article**: Mantsoki, A. *et al.* CpG island erosion, polycomb occupancy and sequence motif enrichment at bivalent promoters in mammalian embryonic stem cells. *Sci. Rep.* **5**, 16791; doi: 10.1038/srep16791 (2015).

## Supplementary Material

Supplementary Information
