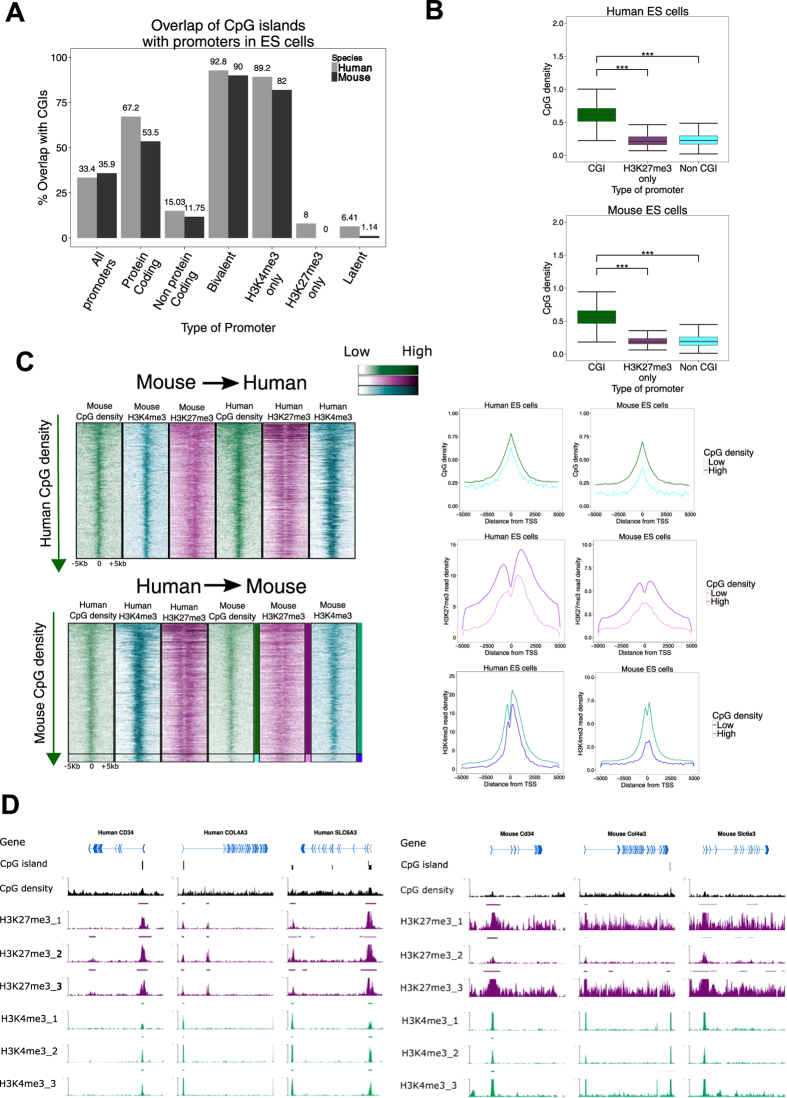# Corrigendum: CpG island erosion, polycomb occupancy and sequence motif enrichment at bivalent promoters in mammalian embryonic stem cells

**DOI:** 10.1038/srep25682

**Published:** 2016-05-23

**Authors:** Anna Mantsoki, Guillaume Devailly, Anagha Joshi

Scientific Reports 5: Article number: 1679110.1038/srep16791; published online: 11192015; updated: 05232016.

This Article contained errors.

In Figure 5c, the labels of the last two panels were inverted, where ‘H3K27me3’ and H3K4me3’ were incorrectly given as ‘H3K4me3’ and ‘H3K27me3’ respectively. The correct Figure 5 appears below as Fig. 1.

In the Supplementary Information file originally published with this Article, Supplementary Tables S12 and S13 were incorrectly labeled as Tables S9 and S10 respectively. In addition, Supplementary Tables S9 and S10 were omitted.

As a result, in the Results section under subheading ‘Bivalent promoters are occupied by fewer transcription factors than active promoters and are specifically enriched in a ‘TCCCC’ sequence motif’.

“The Mzf1 promoter both in mouse and human ES cells is characterized as HC H3K4me3 only and belonged to the low expressed genes in our analysis. However, in recent Mzf1 ChIP-seq experiment performed in HEK293 cell line^51^, the “TCCCC” motif was not enriched in Mzf1 peak list (Table S9)”.

now reads:

“The Mzf1 promoter both in mouse and human ES cells is characterized as HC H3K4me3 only and belonged to the low expressed genes in our analysis. However, in recent Mzf1 ChIP-seq experiment performed in HEK293 cell line^51^, the “TCCCC” motif was not enriched in Mzf1 peak list (Table S12)”.

In the Discussion section,

“We note that H2Aub predominantly but not exclusively marks bivalent promoters (Table S10) as it also marks a fraction of H3K4me3-only expressed gene promoters (Figure S13)”.

now reads:

“We note that H2Aub predominantly but not exclusively marks bivalent promoters (Table S13) as it also marks a fraction of H3K4me3-only expressed gene promoters (Figure S13)”.

These errors have now been corrected in the PDF and HTML versions of the Article, as well as the Supplementary Information that now accompanies the Article.

## Figures and Tables

**Figure 1:**