# Supplementary material for: CpG island erosion, polycomb occupancy and sequence motif enrichment at bivalent promoters in mammalian embryonic stem cells
Source: Sci Rep. 2015 Nov 19;5:16791. doi: 10.1038/srep16791 (PMC4652170; doi:10.1038/srep16791)
Supplement: Supplementary Information [file srep16791-s1.doc]

**Supplementary material for**

**CpG island erosion, polycomb occupancy and sequence motif enrichment at bivalent promoters in mammalian embryonic stem cells**

Anna Mantsoki^1^, Guillaume Devailly^1^ and Anagha Joshi^1^,*

^1^ Division of Developmental Biology, The Roslin Institute and Royal (Dick) School of Veterinary Studies, University of Edinburgh, Easter Bush Campus, Midlothian, EH25 9RG, UK.

* To whom correspondence should be addressed.

Tel: +44 131 651 9100; Fax: +44 131 651 9105; Email: Anagha.Joshi@roslin.ed.ac.uk

**Supplementary methods:**

**Data collection and processing**: Murine ChIP sequencing data for H3K4me3 and H3K27me3 histone marks in ESCs was obtained in fastq format from Gene Expression Omnibus (GEO) database ^1^. Accession numbers for mouse are: SRX001923, SRX085431, SRX122633, SRX172569, SRX266814, SRX266815, SRX305921, SRX305922, SRX001921, SRX185810, SRX122629, SRX172574, SRX266816, SRX266817, SRX305910, and SRX305911. Human ChIP-Seq data (fastq format) for H3K4me3 and H3K27me3 histone marks in hESCs was obtained from Roadmap to epigenomics ^2^ and Gene Expression Omnibus (GEO) ^1^. Accession numbers for human are: SRX006237, SRX012501, SRX027864, SRX007385, SRX019896, SRX006262, SRX006874, SRX012368, SRX007379, SRX019898, SRX003845, SRX064486, SRX027487, SRX189253, SRX027865, SRX056719, SRX003843, SRX064487, SRX027484, SRX189254, SRX040598, SRX056700. After downloading the raw files for all the experiments, each technical and biological replicate of the samples was imported in FastQC 0.10.1 ^3^ for quality control. Alignment of reads was done using Bowtie 0.12.9 ^4^ using reference genomes mm10 for mouse, hg19 for human. For both species, we used single end alignment, seed length=28. We then performed the bowtie execution using custom bash scripts and the samtools ^5^ pipeline to convert sam format file to a bam format file for each sample. The bam files that belonged to the same experiment (technical replicates) were merged into a single bam file in order to proceed with the further analysis. The biological replicates of each experiment were not merged. We downloaded the Gencode ^6^ genes for human (Gencode 19) and mouse (Gencode M2). We created bed files for the promoter regions, keeping the areas that were (-1000 bp, +2000 bp) from the transcription start site (TSS). For mouse, there were 38,922 promoter regions and for human 57,818.

**Peak Calling Method**: We used SICER ^7^ for peak calling, a tool that is recommended for enrichment analysis of histone modification data, since it was shown to outperform other tools developed for performing similar task. The input controls were used when they were provided with the samples. When input was available, the SICER parameters were: for H3K4me3, window=200 and gap size=200. For H3K27me3, window=200 and gap size=2x300, since this histone mark is found covering wider chromatin domains. The rest of the parameters (same for both H3K4me3 and H3K27me3) were effective genome fraction =0.7, false discovery rate (FDR) = 0.01, redundancy threshold = 1 and fragment size = 150. When the control library was unavailable, the FDR value parameter was replaced by the E-value parameter equal to 100. We intersected the resulting files after peak calling with the promoter files using the intersect command from BEDtools ^8^.

**Detection of High Confidence (HC) bivalent, H3K4me3-only, H3K27me3-only and latent promoters**: As mentioned before, we acquired ChIP-seq data (H3K4me3 and H3K27me3) from 8 studies for mouse and 11 studies for human. The resulting files after peak calling were intersected with the promoter files. Our aim was to find whether or not the peaks were overlapping with the regions around the promoters. We used the intersect command from BEDtools ^8^. The resulting bed files for each sample contained the peaks that were found only in promoter regions. In Supplementary Tables 3 and 4, we present the peaks at promoter areas for human and mouse respectively. After the intersection with the promoter areas, we created a matrix that contained the values of all the samples (the rows represent each region and the columns represent the number of peaks that overlap with each region for each sample). For the further analysis, we created R scripts in order to keep the bivalent promoters where both histone mark peaks were identified, the H3K4me3-only promoters where H3K4me3 peak was identified and not H3K27me3, and the H3K27me3-only promoters where only H3K27me3 peak was identified and the latent promoters where peak for neither mark was identified. We obtained all the possible numbers of bivalent regions taking into account combinations for 1 or more data samples until the total numbers of samples (Supplementary tables 5 and 6). The level of stringency was increased as we took into consideration more samples. We defined high-confidence (HC) bivalent promoters as ones identified in 70% or more samples. Therefore, for mouse we would consider a locus as bivalent if it was found in 6 or more samples (6/8 studies) and in human if it was found in 8 or more samples (8/11 studies). We applied the same definition for the H3K4me3-only, H3K27me3-only and latent high-confidence promoters.

**Read density at the promoter regions:** Using BEDtools (8) (coverageBed command) we calculated the coverage at the promoter regions in all different groups for each histone mark sample.

**Peak height and overlap with top peaks:** Using BEDtools ^8^ we intersected the peak files for all the samples in both species with the high-confidence (HC) bivalent regions we had previously detected. We classified the peaks in bivalent and non-bivalent depending on whether they were found or not in HC bivalent promoters. We performed peak height (read density) normalization in each sample then converting it in the logarithmic scale (log10). After checking the significance of the difference of peak height between bivalent and non-bivalent promoters (Student’s t-test), we also checked whether the top peaks of the H3K27me3 samples could give us the same list of HC bivalent promoters. Taking the top high peaks of each H3K27me3 sample, as many as the HC bivalent peaks of the same sample, we checked the degree of overlap between them.

**Functional enrichment analysis**: We conducted gene ontology functional analyses for the bivalent promoters using DAVID ^9^ and AmiGO ^10^.

**Overlap between Species**: To obtain a list of common HC bivalent, H3K4me3-only, H3K27me3-only and latent genes between the species, we used the one2one orthologous regions between human and mouse (16,639 genes from ensembl BioMart) ^11^. We calculated the percentage of conservation for each species individually taking into account the corresponding orthologous regions and their chromatin state for the other species.

**Clustering using published ChIP-seq data:** We downloaded ChIP-seq data from published studies and used them for further classification of our HC bivalent promoters in mouse embryonic stem cells. We gathered four different forms of RNAPII, RNAPIIS5P, RNAPIIS7P and 8WG16 ^12^, PRC2 component, Suz12 ^13^, PRC1 subunits, Cbx7 and Ringb ^13^ in murine ES cells. We also downloaded Jarid2 ^14^, H3K27ac ^15^, Utf1 ^16^ and Ring1b ^17^. We used seqMINER ^18^ to integrate the multiple TFs and histone modifications and visualize the patterns that are formed genome wide at the HC bivalent promoters.

**CpG overlap for the HC promoters:** We calculated the overlap of the HC bivalent, H3K4me3-only, H3K27me3-only and latent regions with the CpG island regions as given from the UCSC tracks CpG islands for hg19 and mm10 ^19^. We calculated the percentage of overlap with the total number of genes for Gencode 19 and Gencode M2, with the protein coding genes and with the all the HC groups we have detected previously in our analysis.

**CpG density and H3K27me3 across species:** We calculated the CpG density as the ratio of observed to expected CpG counts ^20^ for -5Kb, +5kb around the TSS for 100 bp window. The regions we have used were the bivalent regions for each species and their corresponding regions in other species (human/mouse) using the UCSC liftOver tool ^19^. We created heatmaps using custom R scripts for the visualization of the CpG density and H3K27me3 read density ordered by the CpG density of the targeted species.

**Transcription and Epigenetic Factors’ enrichment using published ChIP-Seq data:** We have used data from 49 and 99 ChIP-seq experiments for several transcription factors (TFs), chromatin remodellers and methyltransferases in human and mouse embryonic stem cells respectively ^21^. Initially, we intersected the peak files of all the factors with the promoter regions we have created for the Gencode gene sets. The resulting files were finally intersected with the HC promoters for all the categories and we found the levels of enrichment. For each promoter region we also counted the total number of factors binding significantly at the region. We calculated the numbers of factors binding across the different promoter categories.

**RNA Sequencing levels:** We downloaded the RNA sequencing experiment ^15^ in fastq format. After aligning the reads to the mouse reference genome (mm10) using Bowtie 0.12.9 ^4^, we found the FPKM values using cufflinks 2.2.1 ^22^. For human we used RNA-Seq (FPKM values) data for H1-hESCs from ^23^ . We created three different classes according the expression level. We defined as highly expressed the genes with expression greater than log (FPKM) > 4. Low expression was defined as 0 < log (FPKM) < 4. Finally genes with expression equal to zero belonged to the no expression category.

**Single RNA Sequencing:** Using single cell RNA sequencing data ^24^, we checked the number of genes that had zero levels of expression. We selected genes that belonged to the low expression class (0 < log (FPKM) < 4) for all the HC promoter categories. We then intersected the low expression sets with the FPKM values of the corresponding genes from single RNA-seq. For each gene we counted the number of occurrences of zero expression along the 63 single cell RNA sequencing experiments.

**Gain and loss of function perturbation:**  We collected differentially expressed gene lists (both up- and down-regulated) after over expression of 54 transcription factors and deletion of 37 transcription factors individually in murine ES cells ^25^. We then intersected the gene lists for both gain and loss of function with our HC promoters for all the categories. We checked the levels of perturbation among the promoter types and also which were the genes that were over-perturbed for the majority of the TFs.

**Motif enrichment using HOMER:** We used the gene based analysis with the command findMotifs.pl from HOMER ^26^. We performed the analysis for the HC bivalent and H3K4me3 marked promoters using them both as main and background files to each other. Similarly, the same was applied to all peak list from Najafabadi et al.^27^ C2H2 ChIP-seq experiments.

**P value calculation**: To calculate probability of the overlap of two gene lists can happen due to random chance, we used hypergeometric test. Specifically, to compare two lists we used the phyper function in R. When we were comparing more than two lists we used random permutation of the rows and columns of the results table (species in columns, genes in rows) simulated for 1000 times. We used the permatfull function from the vegan package ^28^ in R. Then we compared the mean of all the simulations with our result of common genes in order to find if there is significant difference between them. We corrected all P values for multiple hypotheses testing using FDR correction.

**Robustness of HC definition:** The number of samples we chose as a cut-off for the detection of HC promoters was 6 for mouse and 8 for human. To validate that the results did not depend on this choice of cut off, we conducted key steps of the analysis for one less and one more samples for both human and mouse. Firstly, we checked the overlap of CpG islands with the HC bivalent, H3K4me3-only, H3K27me3-only and latent promoters. Then, we checked the number of factors binding across the various HC promoter categories and which are the enriched factors for each category. Lastly, we performed de-novo motif discovery using HOMER ^26^.All the analysis is shown in Figure S1 demonstrating the robustness of our findings.

**Supplementary tables:**

Table S1. Accession numbers, type of cell line, growth media and antibodies for the samples gathered for human ES cells (Millipore 07-449 and Upstate 07-449 have no difference)

**Table S2. Accession numbers, type of cell line, growth media and antibodies for the samples gathered for mouse ES cells (Millipore 07-449 and Upstate 07-449 have no difference)**

Table S3. Total reads and peaks detected at promoters for 11 samples of H3K27me3 and H3K4me3 histone modifications in human ES cells

Table S4. Total reads and peaks detected at promoters for 8 samples of H3K27me3 and H3K4me3 histone modifications in mouse ES cells

Table S5. Number of identified promoters in each category as the samples taken into account increase in human ES cells

Table S6. Number of identified promoters in each category as the samples taken into account increase in mouse ES cells

Table S7. Gene ontology terms for the conserved and unique to species HC bivalent genes in Human and Mouse ES cells

Table S8. Factors binding at bivalent, H3K4me3-only and H3K27me3-only promoters in a) human and b) mouse ES cells

Table S9. TCCCC motif enrichment in C2H2 ChIP-sequencing peaks from Najafabadi et al. in Human ES cells. Rank N means that N-1 non TCCCC motif were more enriched.

Table S10. Overlap of HC promoters in all categories with H2Aub1/H3K4me3 promoters. (In total 4518 H2Aub1 peaks where found in promoters and the majority of them was accompanied by H3K4me3)

Table S11. Overlap of validated regions with ChIP-PCR from Mikkelsen et al. with our promoters.

**Supplementary figures:**

| A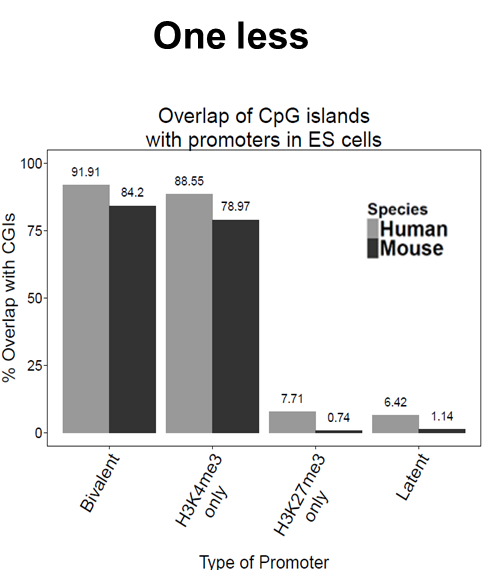 | B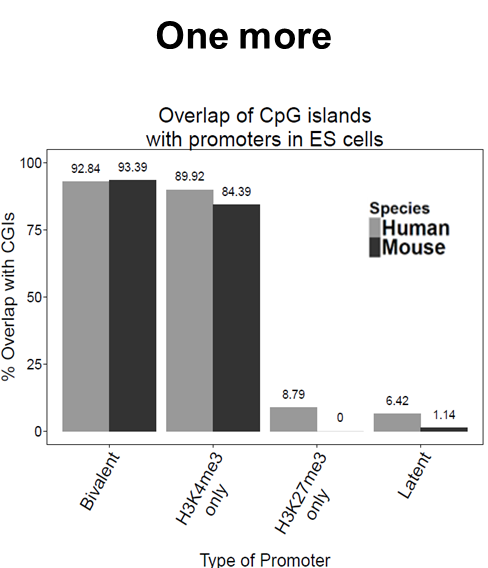 |
| --- | --- |
| 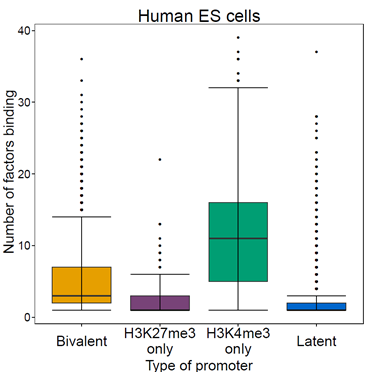 | 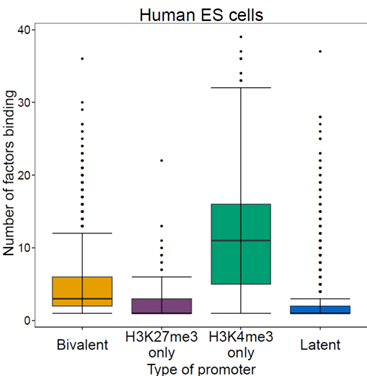 |
| 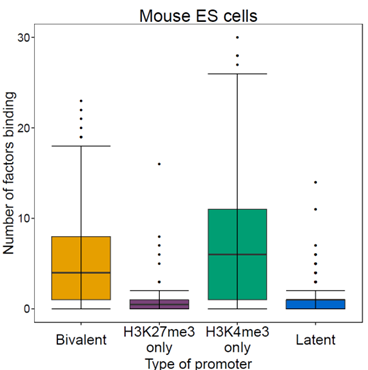 | 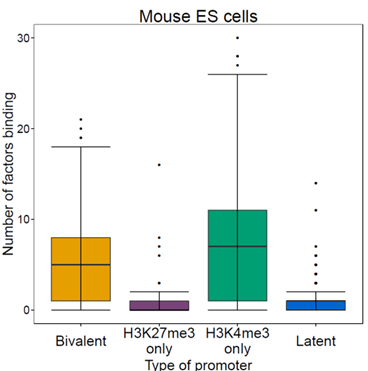 |
| 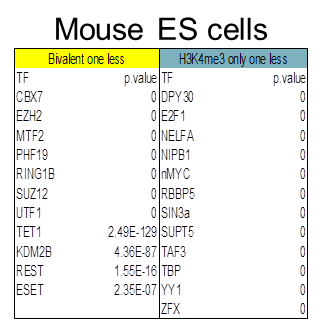 | 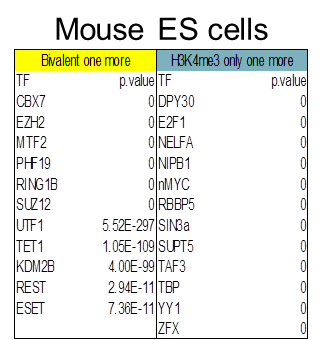 |
| 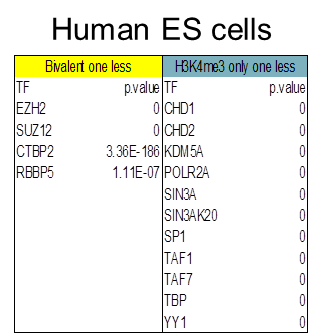 | 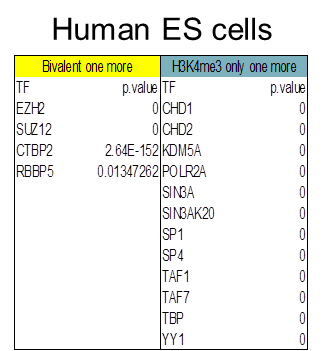 |
| 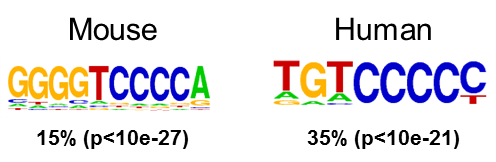 | 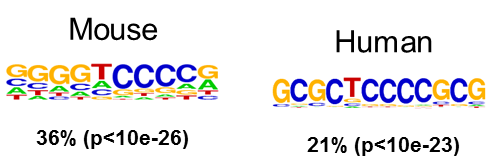 |

Figure S1. Results for CpG enrichment, factor occupancy and factor enrichment remain unchanged when we remove or add one sample from the cut-off.

| a | b |
| --- | --- |
| 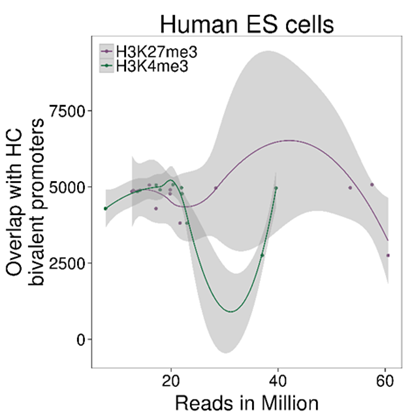 | 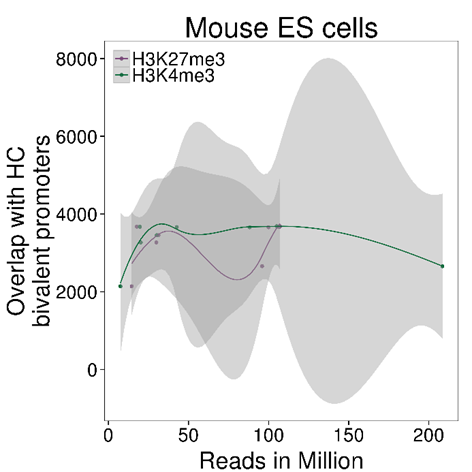 |

Figure S2. Overlap of HC bivalent promoters with bivalent promoters in each sample in a) human and b) mouse ES cells. The correlation of the overlapping HC with the reads of each sample was: For human, r=-0.34 for H3K27me3 samples (purple) and r=-0.38 for H3K4me3. For mouse, r=0.35 for H3K27me3 and r= -0.112 for H3K4me3.

| a | b |
| --- | --- |
| 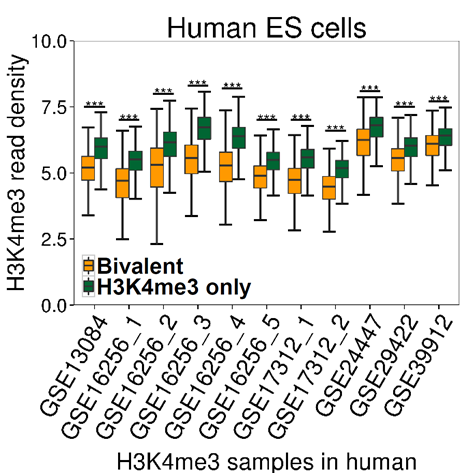 | 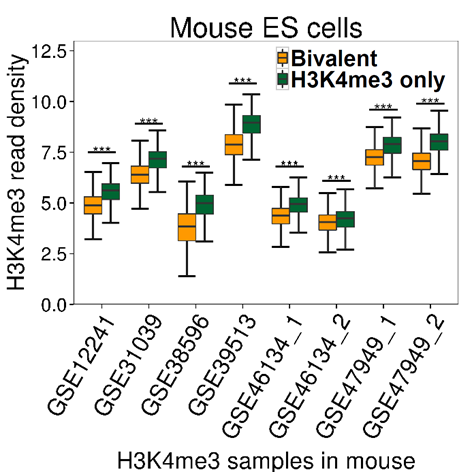 |

Figure S3. H3K4me3 read density at bivalent promoters vs H3K4me3 only promoters in a) human and b) mouse ES cells. (*** P-value<10^-4^)

| **a**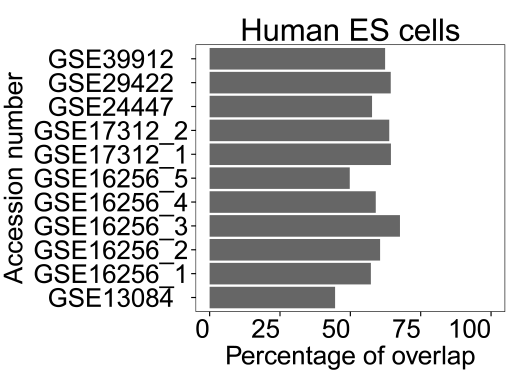 | **b**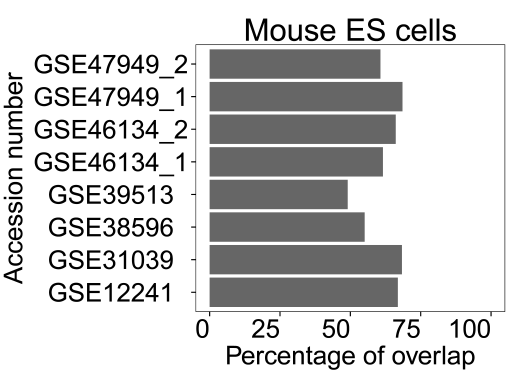 |
| --- | --- |

Figure S4. Overlap of H3K27me3 top promoters with HC bivalent promoters in any individual dataset in a) Human and b) Mouse ES cells

| **a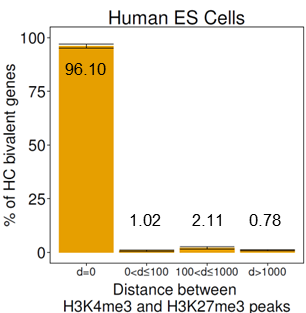** | **b 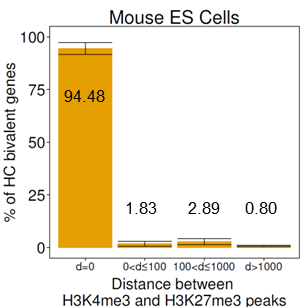** |
| --- | --- |

Figure S5. Mean distance between H3K27me3 and H3K4me3 peaks in all samples in a) Human and b) Mouse ES cells


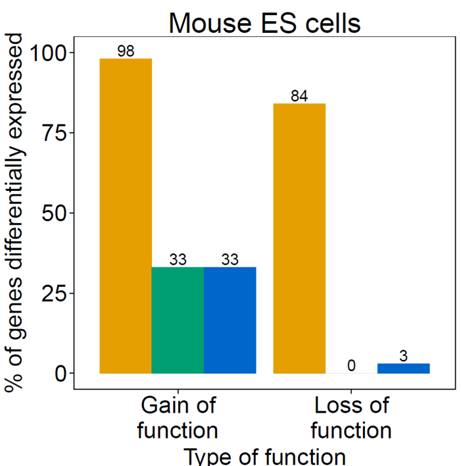


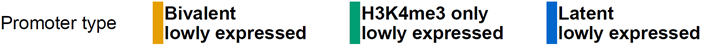


Figure S6. Perturbation of lowly expressed HC bivalent, lowly expressed HC H3K4me3 only and lowly expressed HC latent genes when there is gain or loss of function.

| **a**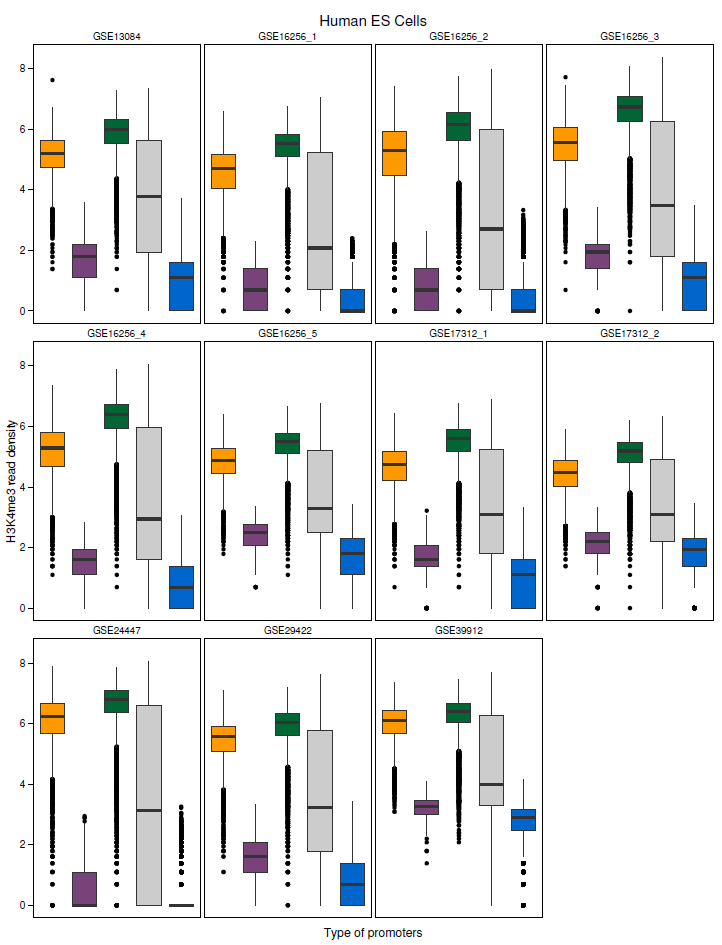 |
| --- |
| **b**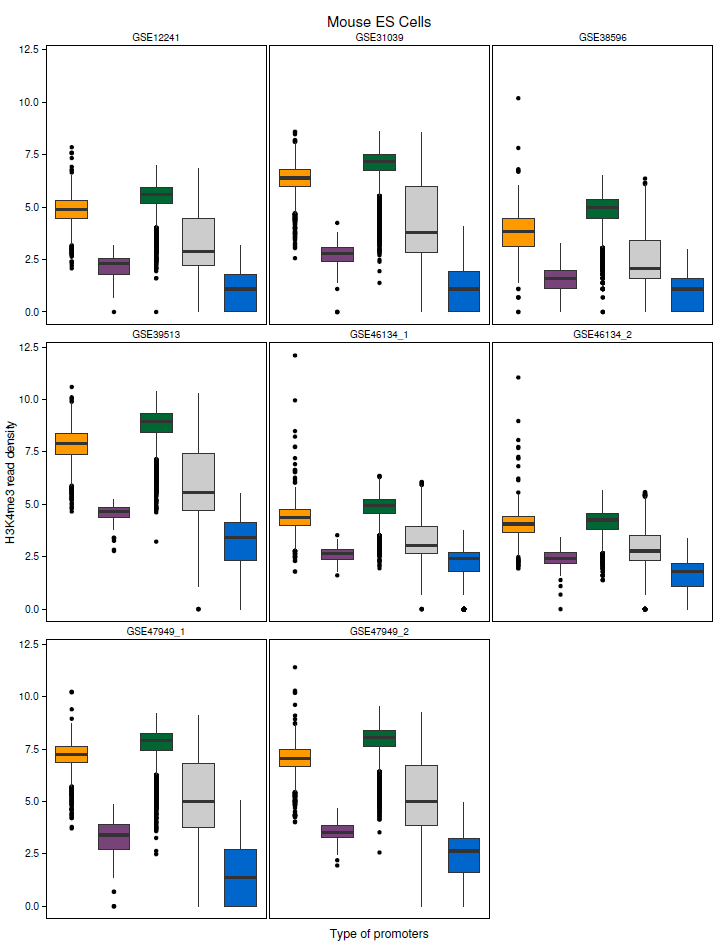 |
| 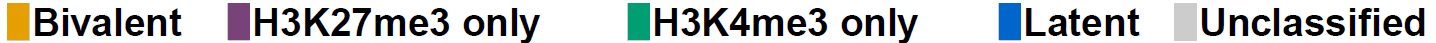 |

Figure S7. Levels of H3K4me3 read density at the promoters according to their classification across samples in a) Human and b) Mouse ES cells

| **a**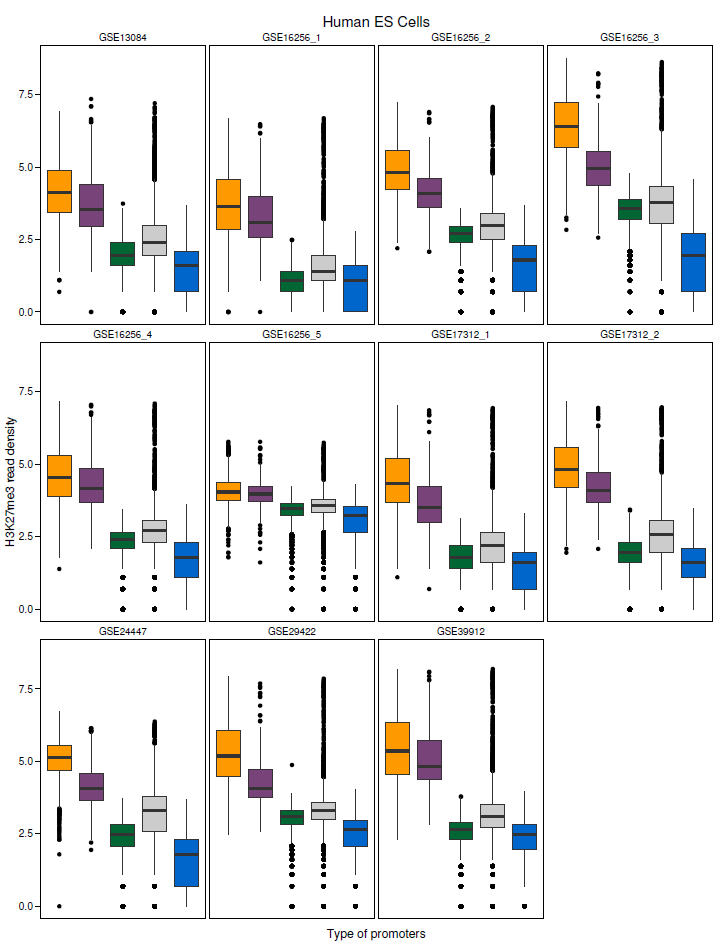 |
| --- |
| **b**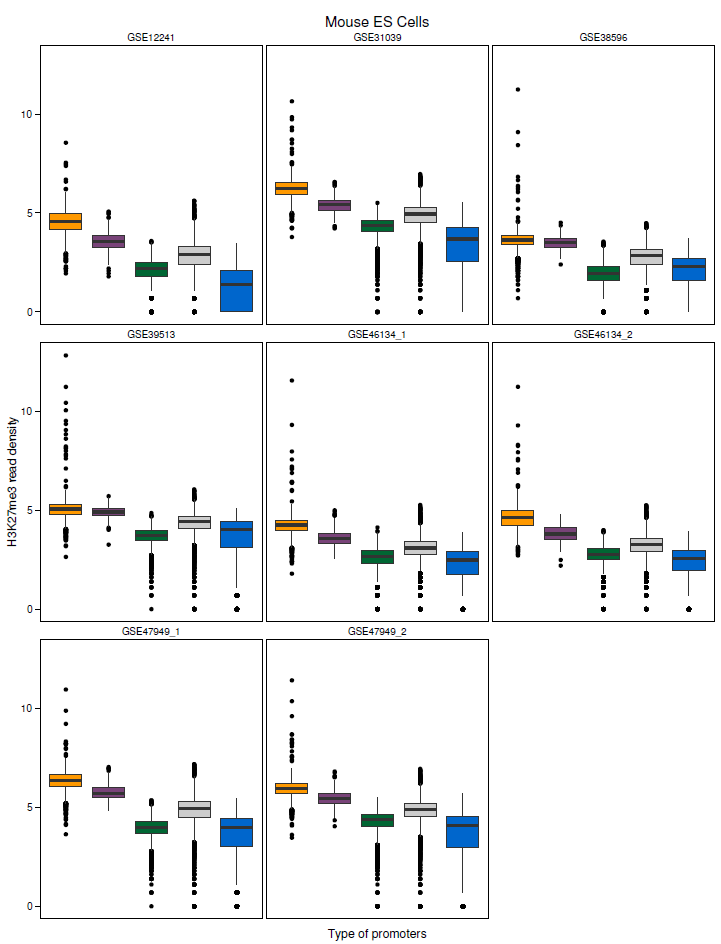 |
| **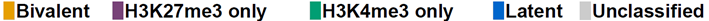** |

Figure S8. Levels of H3K27me3 read density at the promoters according to their classification across samples in a) Human and b) Mouse ES cells


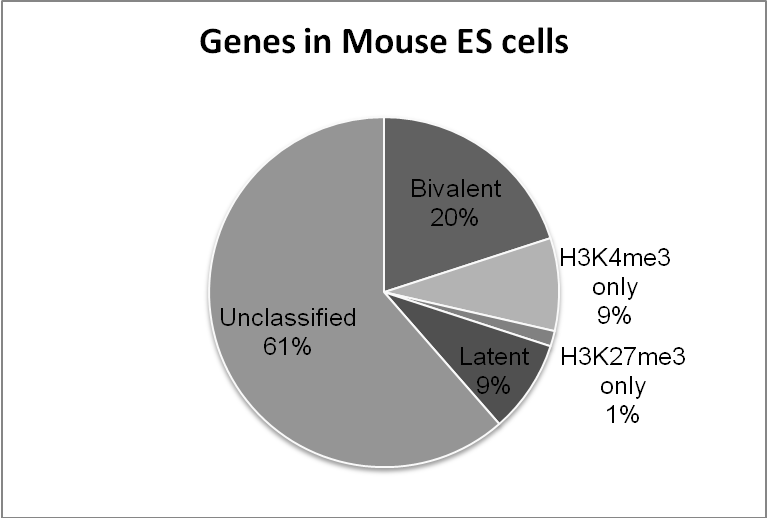


Figure S9. Chromatin status low CpG density promoters in mouse ES cells where corresponding human promoters are CpG-rich and bivalent.

| **a**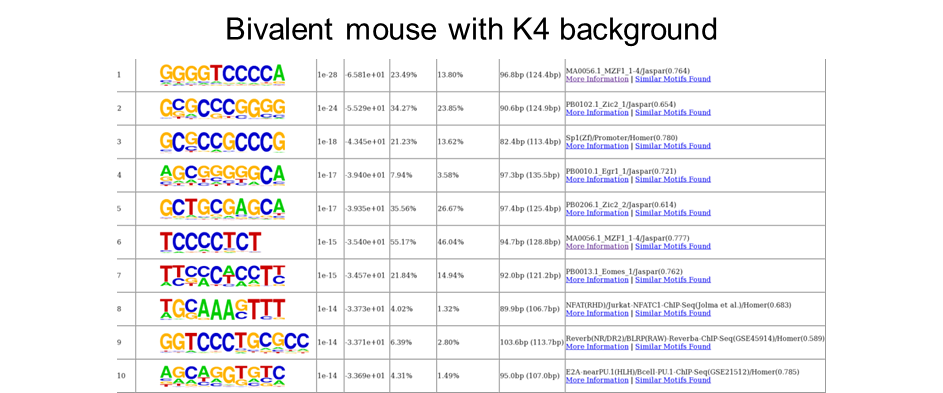 |
| --- |
| **b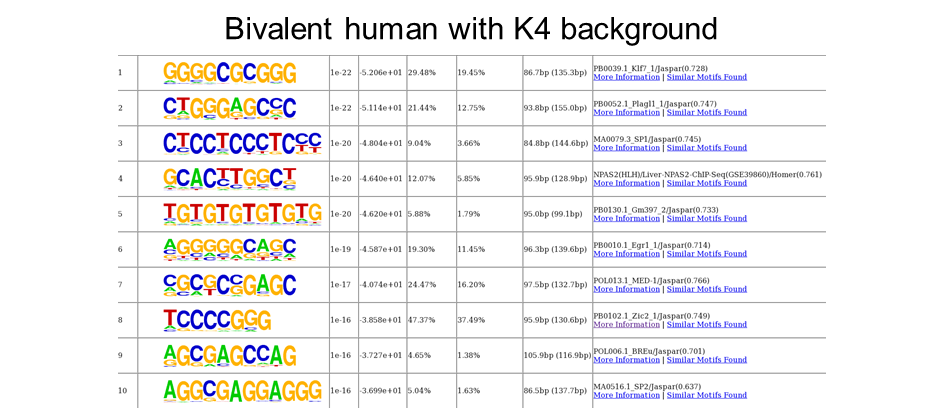** |
| **c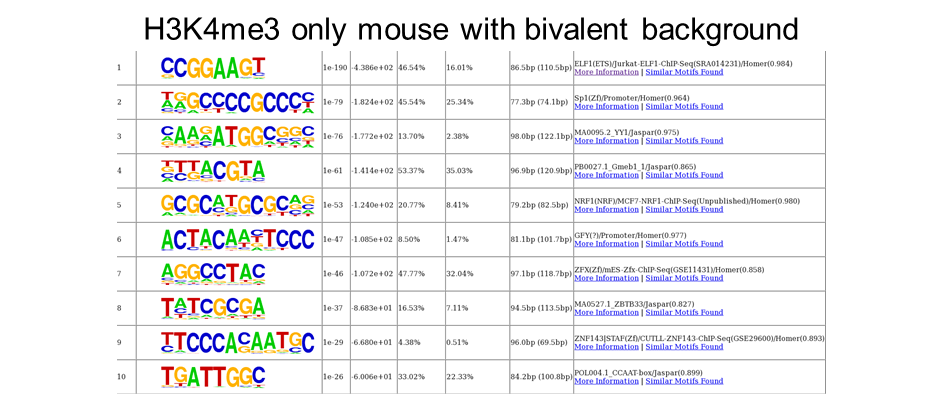** |
| **d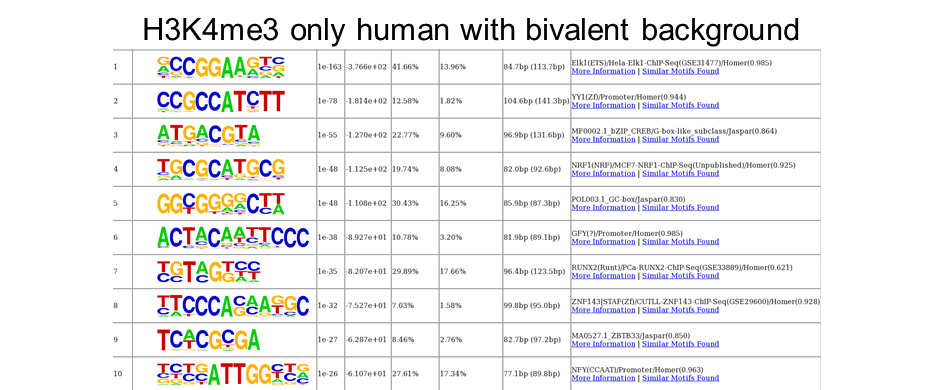** |

Figure S10. De novo motif enrichment for a) bivalent promoters in mouse ES cells, b) bivalent promoters in human ES cells, c) H3K4me3 only promoters in mouse ES cells and d) H3K4me3 only promoters in human ES cells.


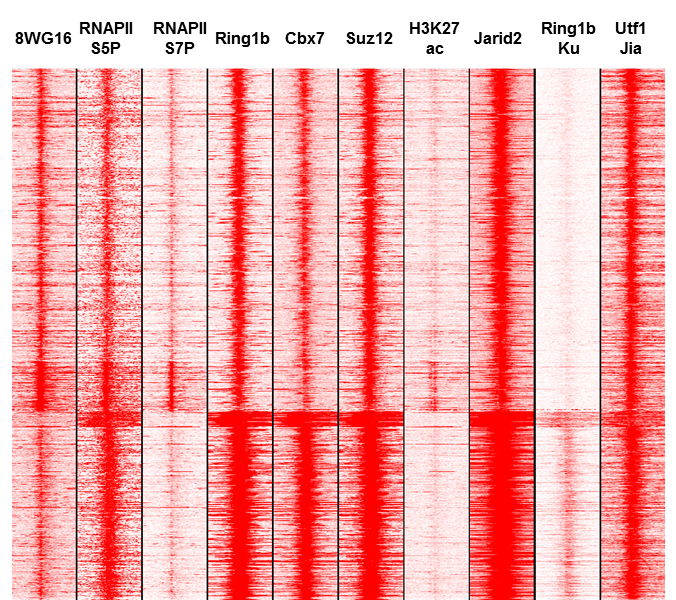


Figure S11. Clustering of HC bivalent promoters in mouse ES cells reveals four different groups of bivalent promoters with either low or high levels of PRC1 (Ring1b). Ring1b-Ku ^17^ sample shown almost no signal in the first two clusters where Ring1b is low. Utf1 ^16^ is present throughout all the clusters.

| 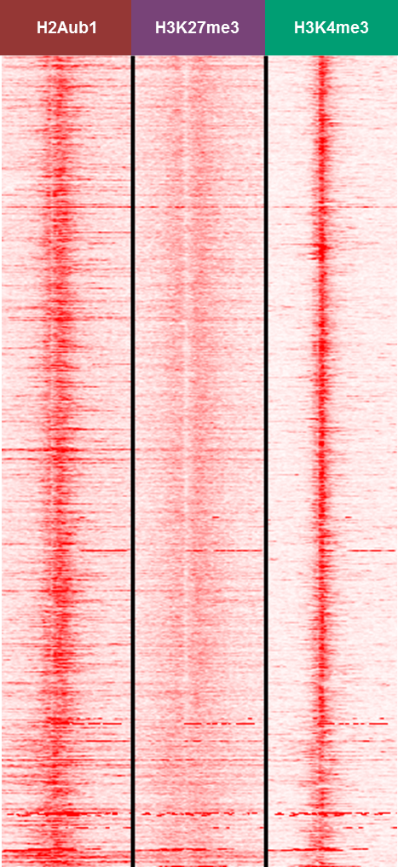 |
| --- |

Figure S12. Signal of H2Aub1, H3K27me3 and H4K4me3 histone modifications at the HC bivalent promoters in mouse ES cells.

| 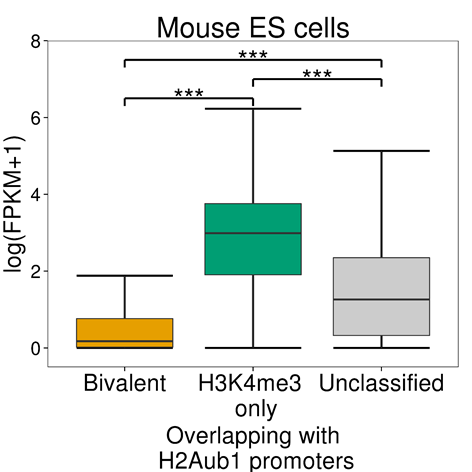 |
| --- |

Figure S13. Expression levels of HC bivalent, H3K4me3 only and Unclassified promoters overlapping with the H2Aub1 bivalent promoters.


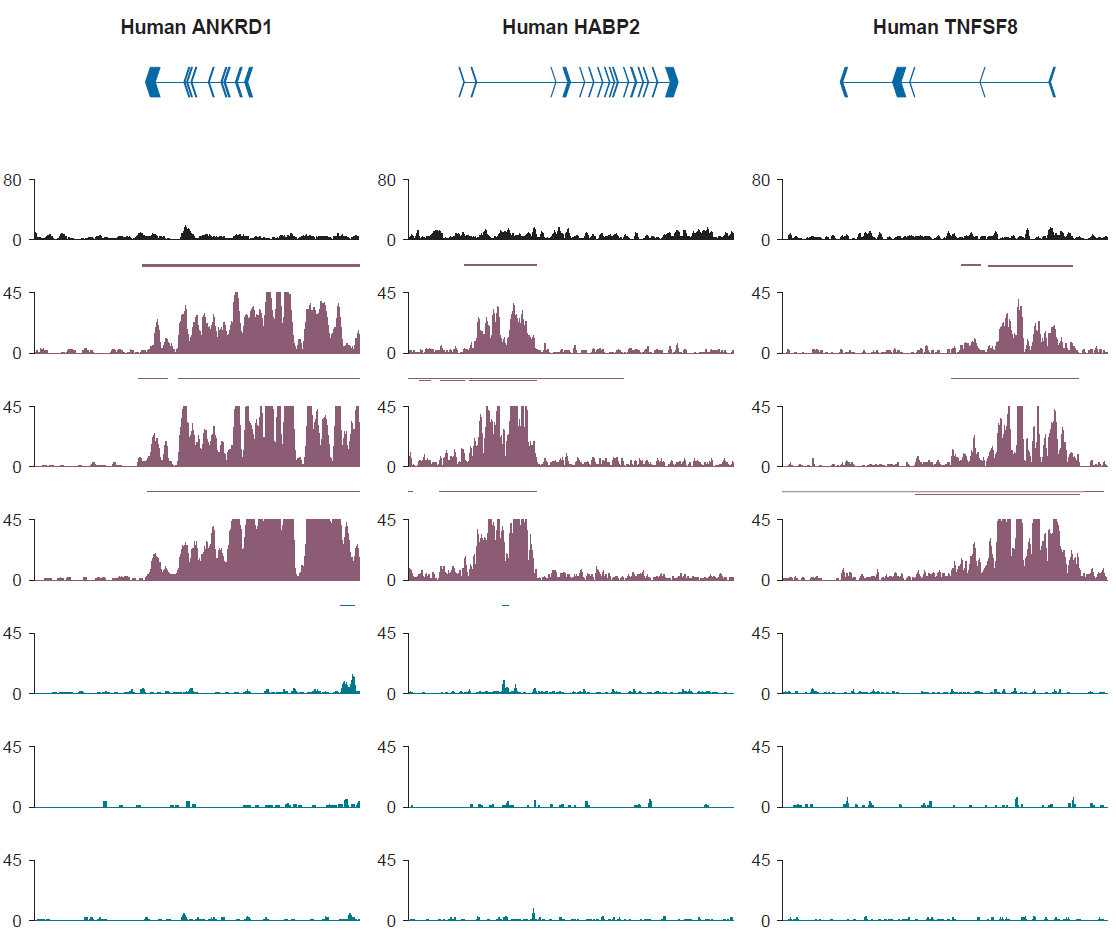


Figure S14. Examples of H3K27me3-only promoters in human ES cells


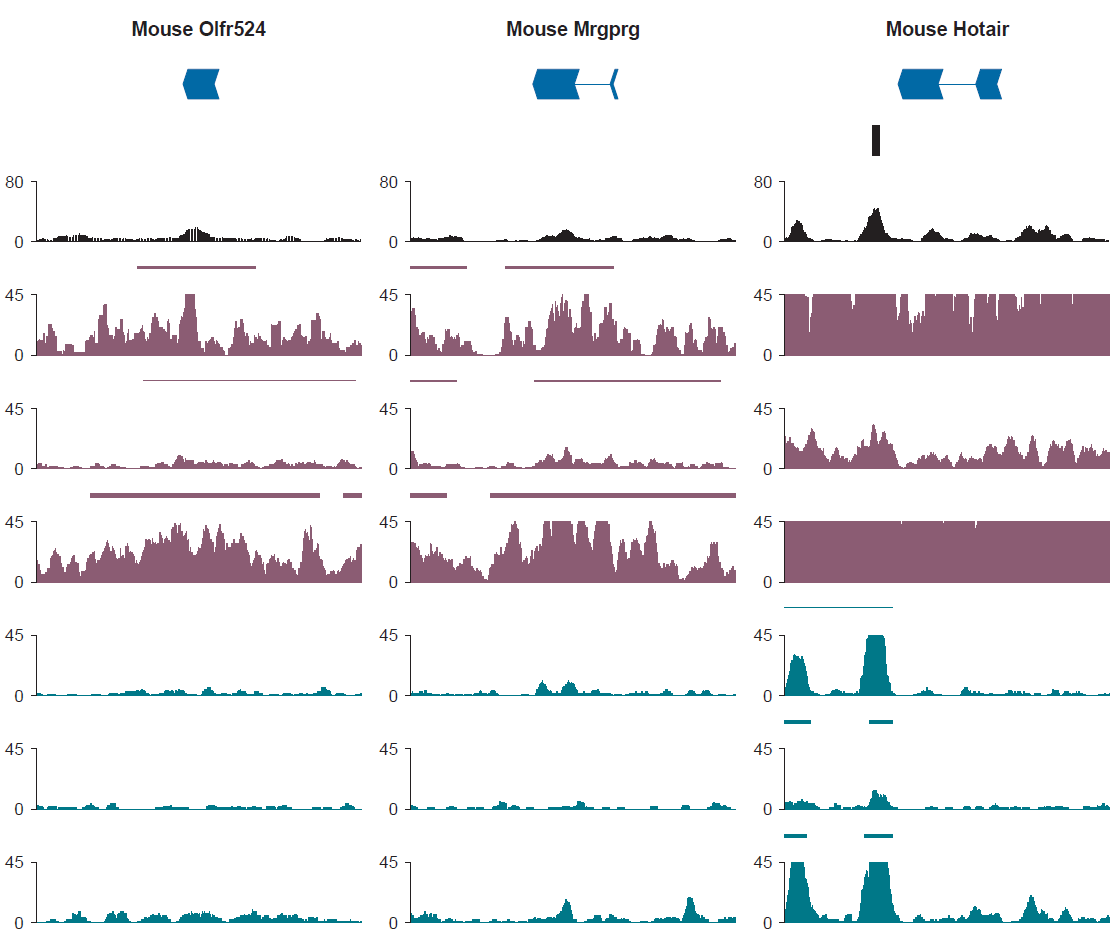


Figure S15. Examples of H3K27me3-only promoters in mouse ES cells

**REFERENCES**

1. Barrett, T., Wilhite, S. E. & Ledoux…, P. {NCBI} {GEO:} archive for functional genomics data sets—update. (2013). doi:10.1093/nar/gks1193

2. Bernstein, B. E. *et al.* The NIH Roadmap Epigenomics Mapping Consortium. *Nat Biotech* **28,** 1045–1048 (2010).

3. S. Andrews. FastQC A Quality Control tool for High Throughput Sequence Data. (2010).

4. Langmead, B., Trapnell, C., Pop, M. & Salzberg, S. L. Ultrafast and memory-efficient alignment of short {DNA} sequences to the human genome. (2009).

5. Li, H., Handsaker, B., Wysoker, A., Fennell, T. & Ruan…, J. The sequence alignment/map format and {SAMtools}. (2009). doi:10.1093/bioinformatics/btp352

6. Harrow, J., Frankish, A., Gonzalez, J. M. & Tapanari…, E. {GENCODE:} The reference human genome annotation for The {ENCODE} Project. (2012).

7. Zang, C. *et al.* A clustering approach for identification of enriched domains from histone modification {ChIP-Seq} data. *Bioinforma. {(Oxford,} England)* **25,** 1952–1958 (2009).

8. Quinlan, A. R. & Hall, I. M. BEDTools: a flexible suite of utilities for comparing genomic features. *Bioinformatics* **26,** 841–2 (2010).

9. Dennis, G. *et al.* DAVID: Database for Annotation, Visualization, and Integrated Discovery. *Genome Biol.* **4,** P3 (2003).

10. Carbon, S. *et al.* AmiGO: online access to ontology and annotation data. *Bioinformatics* **25,** 288–9 (2009).

11. Guberman, J. M. *et al.* BioMart Central Portal: an open database network for the biological community. *Database (Oxford).* **2011,** bar041 (2011).

12. Brookes, E. *et al.* Polycomb associates genome-wide with a specific {RNA} polymerase {II} variant, and regulates metabolic genes in {ESCs.}. *Cell Stem Cell* **10,** 157–170 (2012).

13. Morey, L., Aloia, L., Cozzuto, L., Benitah, S. A. & Di Croce, L. {RYBP} and Cbx7 define specific biological functions of polycomb complexes in mouse embryonic stem cells. *Cell Rep.* **3,** 60–69 (2013).

14. Tee, W.-W., Shen, S. S., Oksuz, O., Narendra, V. & Reinberg, D. Erk1/2 activity promotes chromatin features and RNAPII phosphorylation at developmental promoters in mouse ESCs. *Cell* **156,** 678–90 (2014).

15. Yu, P. *et al.* Spatiotemporal clustering of the epigenome reveals rules of dynamic gene regulation. *Genome Res.* **23,** 352–64 (2013).

16. Jia, J. *et al.* Regulation of pluripotency and self- renewal of ESCs through epigenetic-threshold modulation and mRNA pruning. *Cell* **151,** 576–589 (2012).

17. Ku, M. *et al.* Genomewide analysis of {PRC1} and {PRC2} occupancy identifies two classes of bivalent domains. *{PLoS} Genet.* **4,** (2008).

18. Ye, T. *et al.* seqMINER: an integrated ChIP-seq data interpretation platform. *Nucleic Acids Res.* **39,** e35 (2011).

19. Karolchik, D. *et al.* The UCSC Genome Browser database: 2014 update. *Nucleic Acids Res.* **42,** (2014).

20. Gardiner-Garden, M. & Frommer, M. CpG Islands in vertebrate genomes. *J. Mol. Biol.* **196,** 261–282 (1987).

21. Sánchez-Castillo, M. *et al.* CODEX: a next-generation sequencing experiment database for the haematopoietic and embryonic stem cell communities. *Nucleic Acids Res.* **43,** D1117–23 (2015).

22. Trapnell, C. *et al.* Differential gene and transcript expression analysis of RNA-seq experiments with TopHat and Cufflinks. *Nat. Protoc.* **7,** 562–78 (2012).

23. Djebali, S. *et al.* Landscape of transcription in human cells. *Nature* **489,** 101–8 (2012).

24. Streets, A. M. *et al.* Microfluidic single-cell whole-transcriptome sequencing. *Proc. Natl. Acad. Sci. U. S. A.* **111,** 7048–53 (2014).

25. Xu, H. *et al.* ESCAPE: database for integrating high-content published data collected from human and mouse embryonic stem cells. *Database (Oxford).* **2013,** bat045 (2013).

26. Heinz, S. *et al.* Simple combinations of lineage-determining transcription factors prime cis-regulatory elements required for macrophage and B cell identities. *Mol. Cell* **38,** 576–89 (2010).

27. Najafabadi, H. S. *et al.* C2H2 zinc finger proteins greatly expand the human regulatory lexicon. *Nat. Biotechnol.* (2015). doi:10.1038/nbt.3128

28. Oksanen, J. *et al.* vegan: Community Ecology Package. (2013).
